# Endoplasmin Is a Hypoxia-Inducible Endoplasmic Reticulum-Derived Cargo of Extracellular Vesicles Released by Cardiac Cell Lines

**DOI:** 10.3390/membranes13040431

**Published:** 2023-04-13

**Authors:** Anna Koncz, Lilla Turiák, Krisztina Németh, Dorina Lenzinger, Tünde Bárkai, Péter Lőrincz, Helga Zelenyánszki, Krisztina V. Vukman, Edit I. Buzás, Tamás Visnovitz

**Affiliations:** 1 Department of Genetics, Cell and Immunobiology, Semmelweis University, Üllői út 26, 1085 Budapest, Hungary; 2 Research Centre for Natural Sciences, Institute of Organic Chemistry, Magyar Tudósok Körútja 2, 1117 Budapest, Hungary; 3 ELKH-SE Translational Extracellular Vesicle Research Group, Nagyvárad tér 4, 1085 Budapest, Hungary; 4 Department of Anatomy, Cell and Developmental Biology, ELTE Eötvös Loránd University, Pázmány Péter sétány 1/c, 1117 Budapest, Hungary; 5 Department of Plant Physiology and Molecular Plant Biology, ELTE Eötvös Loránd University, Pázmány Péter sétány 1/c, 1117 Budapest, Hungary; 6 HCEMM-SU Extracellular Vesicle Research Group, Nagyvárad tér 4, 1085 Budapest, Hungary

**Keywords:** cardiomyocyte, extracellular vesicle, exosome, small EV, medium EV, endoplasmic reticulum stress

## Abstract

Cardiomyopathies are leading causes of human mortality. Recent data indicate that the cardiomyocyte-derived extracellular vesicles (EVs) released upon cardiac injury are present in circulation. This paper aimed to analyze EVs released under normal and hypoxic conditions by H9c2 (rat), AC16 (human) and HL1 (mouse) cardiac cell lines. Small (sEVs), medium (mEVs) and large EVs (lEVs) were separated from a conditioned medium by a combination of gravity filtration, differential centrifugation and tangential flow filtration. The EVs were characterized by microBCA, SPV lipid assay, nanoparticle tracking analysis, transmission and immunogold electron microscopy, flow cytometry and Western blotting. Proteomic profiles of the EVs were determined. Surprisingly, an endoplasmic reticulum chaperone, endoplasmin (ENPL, grp94 or gp96), was identified in the EV samples, and its association with EVs was validated. The secretion and uptake of ENPL was followed by confocal microscopy using GFP-ENPL fusion protein expressing HL1 cells. We identified ENPL as an internal cargo of cardiomyocyte-derived mEVs and sEVs. Based on our proteomic analysis, its presence in EVs was linked to hypoxia in HL1 and H9c2 cells, and we hypothesize that EV-associated ENPL may have a cardioprotective role by reducing cardiomyocyte ER stress.

## 1. Introduction

Cardiovascular diseases are leading causes of morbidity and mortality globally, especially in Western countries. Recently, we have demonstrated that cardiomyocyte-derived extracellular vesicles (EVs), released upon cardiac injury, are detectable in the blood plasma [1] and thus may be potentially used as circulating biomarkers of cardiac damage. EVs are small particles, surrounded by a phospholipid bilayer, that are released by all known cells. In addition to their crucial role in the maintenance of cellular homeostasis, EVs convey information between cells, and they have attracted substantial interest in biomedicine [2,3,4,5,6,7]. Principally, EVs are categorized based on their subcellular origin [5,8]. However, currently there are no established molecular markers available for the different biogenesis routes; therefore, operational classification based on biophysical or biochemical parameters (such as size) is suggested [5]. EVs of endosomal or amphisome origin are called exosomes [5,8,9]. Recent studies have confirmed that sEVs are also formed via plasma membrane budding [8,9,10]. Ectosomes, which are shed from the plasma membrane [8,9], include microvesicles with a diameter range of 200 to 800 nm [3] and larger ectosomes (lEVs) such as apoptotic bodies, oncosomes and exophers. Additionally, MVB-like sEV clusters released *en bloc* are also produced with a diameter ≥ 1 µm [8,9,11]. The MISEV (Minimal Information for Studies of Extracellular Vesicles) guidelines (MISEV2014 and MISEV2018) are important results of the EV research community’s standardization efforts [4,5].

Endoplasmin (ENPL), also referred to as glucose-regulated protein 94 (grp94, gp96 or HSP90B1), is encoded by the *HSP90B1* gene and is primarily localized to the endoplasmic reticulum (ER) [12]. It is present in the lumen of the ER and the sarcoplasmic reticulum of muscle cells without any preferential association with the membranes of the ER. It is occasionally also secreted by cells, and under special conditions, it can be found on the outer surface of the plasma membrane [13]. In the ER, ENPL directs the folding of some secreted and membrane proteins, such as intermediates of the light chains of immunoglobulins or insulin-like proteins (IGF-I and IGF-II) and Toll-like receptors [14]. In addition to its chaperone function, ENPL is one of the calcium-binding proteins of the ER lumen and plays a crucial role during ER stress [15]. The activity of ENPL is essential for life: homozygote grp94-/-mouse embryos die on day 7 of gestation. Importantly, ENPL takes part in the normal differentiation of cardiac, smooth and skeletal muscle cells [16]. In addition, previous research has demonstrated cardioprotective effects of ENPL in cell lines (C2C12 and H9c2), in a goat model and in human cardiomyocytes. The levels of ENPL were found to be increased significantly and improved the survival of cardiomyocytes in fibrillating atria [17], under ER stress [18] and during hypoxia treatment [19,20].

Hypoxic conditions may induce changes in EV cargo molecules [21]. In vitro primary rat cardiomyocytes release EVs containing heat shock proteins (such as HSP60, HPS70 and HSP90) to alert surrounding cells [22,23]. It was shown that in various tumor types, hypoxia causes dramatic changes in the EV cargo miRNAs and proteins [21]. Although the cytoplasmic (HSP70 and HSP90) and mitochondrial resident HPS60 are well-known EV cargo proteins, the endoplasmic-reticulum-derived proteins are listed among non-EV contaminants of EV preparations by the MISEV2018 guideline and are recommended as negative controls during Western blotting and flow cytometry [5].

Remarkably, we found ENPL, a stress-inducible, ER-resident molecular chaperon protein in cardiomyocyte-derived EVs. Furthermore, we provided evidence that it is an internal cargo molecule of cardiomyocyte-derived EVs rather than an ER-derived purification artefact. We hypothesize that the release of ENPL by EVs may represent a chaperone-mediated cardioprotective mechanism for the maintenance of cardiomyocyte homeostasis.

## 2. Materials and Methods

### 2.1. Cell Culture

The cell lines used in this study included the AC16 human (Merck, New York, NY, USA) and the H9c2 (2-1) BDIX rat (ECACC, Salisbury, UK) cardiomyoblast cell lines and the HL1 immortalized mouse cardiomyocyte cell line (Merck, New York, NY, USA). The cell lines were cultured as previously described [7,24]. Prior to EV separation, H9c2 and AC16 cells were differentiated according to the published protocols [24,25,26]. In the case of the H9c2 cells, upon reaching 100% confluence, the FBS concentration was reduced to 1% for 24 h, and the cells were further cultured and differentiated with 1% FBS and 10 nM retinoic acid (Sigma, Darmstadt, Germany) for an additional 6 days. In case of AC16, during the differentiation, the DMEM/F12 medium (Gibco, New York, NY, USA) was supplemented with 2% horse serum and 1% Insulin-Transferrin-Selenium (both Gibco). The AC16 cells were used for the experiments after 7 days of differentiation. The cell cultures were regularly monitored for Mycoplasma infections by PCR, using the forward GAAGAWATGCCWTATTTAGAAGATGG and the reverse CCRTTTTGACTYTTWCCACCMAGTGGTTGTTG primers.

### 2.2. EV Separation from Conditioned Cell Culture Medium

Conditioned-medium-derived EVs were separated as previously described, with some modifications [7]. Briefly, the cells were placed into a serum-free medium for 16 h either under normoxia or hypoxia to avoid EVs derived from the FBS [5]. Hypoxia was achieved using an Oxoid AnaeroGen 2.5L Sachet (ThermoFisher, Waltham, MA, USA). The cell viability was assessed by a trypan blue dye (Gibco, New York, NY, USA) exclusion test and a Resazurin Cell Viability Assay Kit (Biotium, Fremont, CA, USA). The viability data are shown in Supplementary Tables S1 and S2. The 16 h long serum starvation did not affect the cell viability. Size-based EV subpopulations (including lEVs, mEVs and sEVs) were separated by the combination of gravity-driven filtration and differential centrifugation [7]. Before ultracentrifugation, tangential flow filtration (TFF Easy, 20 nm pore size, HansaBioMed, Tallinn, Estonia) was applied [27]. The mEV and sEV pellets were resuspended in PBS, HPLC clean, distilled water or RIPA lysis buffer before further investigation. The EVs were aliquoted, snap-frozen in liquid nitrogen and stored at −80 °C until further use.

### 2.3. Determination of Protein and Lipid Contents of EVs

The purity of the EV fractions was assessed by measuring the total protein and total lipid contents [28]. The protein content of the EVs was determined using a microBCA Protein Assay kit (ThermoFisher, Waltham, MA, USA), according to the instructions of the manufacturer. The total lipid content was measured using a Lipid Detection Kit (Bioxol, Budapest, Hungary) as previously described [7]. The serum-free Claycomb medium (Merck, Darmstadt, Germany) of the HL1 cells contains a high concentration of proteins (e.g., bovine albumin) [29]; consequently, significant amounts of proteins may adsorb onto the EV surface as part of their protein corona [30,31]. Due to this unknown external protein cargo, we decided to standardize the HL1-derived EV preparations based on their lipid content only. Samples were excluded from further investigations based on an arbitrarily determined total lipid lower threshold of 1 µg.

### 2.4. Nanoparticle Tracking Analysis (NTA)

The EV samples were diluted to 1mL in PBS, and both particle concentration and size distribution were measured using a ZetaView PMX120 NTA instrument (Particle Metrix GmbH, Inning am Ammersee, Germany) For each measurement, 11 positions (2 cycles per position) were scanned at 25 °C. The following settings were used: auto expose; shutter speed—100; gain—28.8; offset—0; sensitivity—80 for sEVs and 75 for mEVs; frame rate—30 for sEVs and 7.5 for mEVs. The recorded videos were analyzed with a minimum area of 5, a maximum area of 1000 and a minimum brightness of 20 by ZetaView Analyze software 8.05.10 (Particle Metrix GmbH, Inning am Ammersee, Germany) [32]. When comparing the EV release from the different experimental groups, the cells were cultured under identical conditions (medium volume; tissue culture flask surface area).

### 2.5. Mass Spectrometry (MS)

A proteomic analysis by mass spectrometry was carried out as described previously [30]. EV pellets (sEV and mEV) were suspended in 20 µL HPLC clean, distilled water, and samples were stored at −80 °C until analysis. The EVs were lysed, and the proteins were solubilized by repeated freeze–thaw cycles and digested as previously described [33]. For sample analysis, a Dionex Ultimate 3000 nanoRSLC (Dionex, Sunnyvale, CA, USA) coupled to a Bruker Maxis II mass spectrometer (Bruker Daltonics GmbH, Bremen, Germany) via CaptiveSpray nanobooster ion source was used. The peptides were separated on an ACQUITY UPLC M-Class Peptide BEH C18 column (130 Å, 1.7 µm, 75 µm × 250 mm, Waters), following trapping on an Acclaim™ PepMap100™ C18 NanoTrap column (100 Å, 5 µm, 100 µm × 20 mm, ThermoFisher, Waltham, MA, USA). For protein identification search engines, Mascot (Matrix Science, Boston, MA, USA) and X!Tandem (GPM, Winnipeg, MB, Canada) were used against the Swissprot *Homo sapiens*, *Rattus norvegicus* and *Mus musculus* database. The data were analyzed with the help of Scaffold 4.5.3 (Proteome Software, Inc., Portland, OR, USA) programs. Venn diagrams were created with the help of http://bioinformatics.psb.ugent.be/webtools/Venn/ accessed on 13 December 2021.

### 2.6. Detection of EVs by Transmission Electron Microscopy (TEM)

Whole-mounted sEVs and mEVs were visualized as described by Théry et al. [34]. For immunogold TEM, we used a combination of the previously described methods with minor modifications [7,34]. A total of of 5 µL of EV suspension was placed on formvar-coated Ni grids (SPI Supplies, West Chester, PA, USA), and after 10 min of incubation at room temperature (RT), the excess liquid was removed using filter paper. The EVs were fixed by 4% paraformaldehyde (PFA) for 10 min at RT and washed 3 times for 5 min with distilled water. For blocking, a 2% sucrose (Molar Chemicals, Halásztelek, Hungary) solution was used in PBS for 1 h at RT [35]. The primary antibodies were applied in a 2% sucrose solution in PBS overnight at 4 °C. Following three 5 min washes at RT with a 2% sucrose solution, secondary antibodies were applied in 2% sucrose for 1 h at RT. We used 2% sucrose instead of bovine serum albumin (BSA) to avoid non-specific protein binding to the formvar coat and non-specific immunoreactivity to the BSA. Unbound secondary antibodies were removed by three washes (for 5 min at RT), and any residual sucrose was removed by washing with PBS (3 times for 5 min at RT). The samples were post-fixed using 2% glutaraldehyde (Serva Electrophoresis GmbH, Heidelberg, Germany) and washed with distilled water 3 times for 5 min at RT. Following immunogold labelling, positive–negative contrasting was performed [34]. The samples were examined by a JEOL 1011 transmission electron microscope (JEOL, Tokyo, Japan). The diameters of different EVs were determined by the ImageJ software.

For the detection of the EV marker CD63 [5], a mouse anti-CD63 IgG antibody (Santa Cruz Biotechnology, Dallas, TX, USA) was applied. ENPL was detected by rabbit anti-GRP94 polyclonal antibody (Merck, Darmstadt, Germany). As secondary antibodies, polyclonal goat anti-rabbit IgG 10 nm gold (Sigma, Darmstadt, Germany) or polyclonal goat anti-mouse IgG 5 nm gold (Sigma, Darmstadt, Germany) were used. All antibodies used in these experiments are listed in Supplementary Table S3.

### 2.7. Flow Cytometry

The expression of EV markers, CD63, CD81, ALCG-2-interacting protein X (ALIX), tumor-susceptibility gene 101 protein (TSG101) [5] and ENPL were investigated. AC16-, H9c2-, and HL1-derived mEVs and sEVs bound to latex beads were examined by flow cytometry. Particles (2 × 10^8^) were bound onto the surface of latex beads (4 µm, Aldehyde/Sulfate Latex Beads, ThermoFisher, Waltham, MA, USA), overnight at 4 °C with rotation (10 rpm, Fisherbrand™ MiniTube Rotator, ThermoFisher, Waltham, MA, USA). EVs were fixed with 4% PFA. The excess fixative was removed by centrifugation (1000× *g*, 10 min). The free surface of the latex beads was blocked with 300 mM glycine (Sigma, Darmstadt, Germany). We used 10% BSA (Serva Electrophoresis GmbH, Heidelberg, Germany) supplemented with 0.3% TritonX-100^®^ (Molar Chemicals, Halásztelek, Hungary) in the case of intravesicular staining (ALIX, TSG101 and ENPL). After washing with PBS (1000 g, 10 min), the latex beads were incubated in PBS containing 5% BSA with the primary antibodies for 30 min at RT. The following primary antibodies were used at 1:500 dilutions: anti-human CD63, anti-human CD81, anti-human grp94, anti-human TSG101 and anti-human ALIX. Excess antibodies were removed by centrifugation (1000× *g*, 10 min), and the latex beads were incubated with secondary antibodies in PBS containing 1% BSA for 30 min at RT. Anti-rabbit ATTO488 and anti-mouse CF488 secondary antibodies (Sigma, Darmstadt, Germany) were used at a 1:1000 dilution. Between labelling steps, the samples were washed with PBS. Antibody control samples were prepared by blocking the surface of the latex beads (without EVs) with 100 mM glycine and 2.5% BSA solution. Afterwards, the beads were incubated with antibodies. Following a centrifugation step, 5000 events/sample were measured using a CytoFLEX S flow cytometer (Beckman Coulter, Brea, CA, USA). The data were analyzed using FlowJo_V10 software (BD, Franklin Lakes, NJ, USA). Details of the antibodies used are provided in Supplementary Table S3.

To compare the total number of EVs produced by different cell lines (HL1, H9c2 and AC16) under hypoxia and control conditions, equal numbers of cells were cultured in T25 flasks for 16 h in serum-free media, and the conditioned and cell-free supernatants were collected. Subsequently, the remaining cells and lEVs were eliminated by centrifugations at 4 °C of 10 min and 300 g and 30 min and 2000 g. The phospholipid membranes of the EVs were stained with the BioxMLRed (Bioxol, Budapest, Hungary) lipophilic and auto-quenchable dye and measured immediately with an Apogee flow cytometer. The instrumental settings were adjusted with ApogeeMix (#1527) and APC-MESF beads (BD, Franklin Lakes, NJ, USA). Particles were assessed as EVs if they were lipid staining positive and showed detergent sensitivity when exposed to 0.1% of TritonX-100^®^ (Molar Chemicals, Halásztelek, Hungary). The data were analyzed using FlowJo_V10 software (BD, Franklin Lakes, NJ, USA).

### 2.8. Western Blotting

When the purified EVs were used for qualitative Western blotting, the EV pellets were suspended and lysed in RIPA buffer with cOmplete Protease Inhibitor Cocktail (Roche, Basel, Switzerland). A reliable loading control for the purified EVs for Western blotting is currently lacking [5], and during the EV purification, the uniformity of the samples could not be guaranteed. Thus, we performed quantitative Western blots of conditioned media from an equal number of cultured cells. We analyzed the total proteins of the serum-, cell- and lEV-free supernatants. Proteins of the conditioned media were precipitated as previlusly described [36]. Following subsequent three acetone washes, the protein pellets were re-suspended in RIPA buffer with complete Protease Inhibitor Cocktail (Roche, Basel, Switzerland). Polyacrylamide gel electrophoresis [37] was performed using 10% gels with an acrylamide/bis-acrylamide ratio of 37.5:1 (Serva Electrophoresis GmbH, Heidelberg, Germany) and a MiniProtean (BioRad, Hercules, CA, USA) gel-running system. To increase the efficiency of membrane protein solubilization, a mixture of 0.1% TritonX^®^-100 (Molar Chemicals, Halásztelek, Hungary) and Laemmli buffer was used as previously described [38]. Approximately 4 µg protein was loaded per well. After separation, the proteins were transferred to the PVDF membrane (Serva Electrophoresis GmbH, Heidelberg, Germany). A blocking solution (5% skimmed milk powder in washing buffer) was used for 1 h at RT. Primary antibodies, including rabbit anti-ALIX, rabbit anti-TSG101, rabbit anti-CD63, rabbit anti-CD81, rabbit anti-grp94 (all from Merck, Darmstadt, Germany) and antibodies to ENPL (ThermoFisher, Waltham, MA, USA) were applied in a 1:1000 dilution. A 1:2000 dilution of anti-N terminal β-actin (Sigma, Darmstadt, Germany) in a blocking solution was used as a positive control overnight at 4 °C. A peroxidase-labeled anti-rabbit secondary antibody (abcam, Cambridge, UK) was used in a 1:10,000 dilution. Chemiluminescent signals were detected by an ECL Western Blotting Substrate (ThermoFisher, Waltham, MA, USA) with an Imager CHEMI Premium (VWR, Radnor, PA, USA) image analyzer system. Details of the antibodies are listed in Supplementary Table S3. Densitometry was performed using the ImageJ software.

### 2.9. GFP-ENPL Fusion Protein Expression

#### Generation of pMAXGFP-ENPL Plasmid

Amino acid sequences of rat (Q66HD0) and mouse (P08113) ENPL were obtained from the UniProt database (https://www.uniprot.org/). The sequences were fit by Clustal Omega (https://www.ebi.ac.uk/Tools/msa/clustalo/) and Protein Blast (https://blast.ncbi.nlm.nih.gov/Blast.cgi) accessed on 3 January 2020. Altogether, a similarity of 98.01% was found between the ENPL primary amino acid sequences in the two species (Supplementary Figure S1). The Q66HD0 rat ENPL sequence was used in our work. Based on the protein sequence, codon optimization was performed with Codon Optimization Tool of Integrated DNA Technologies, Inc. (https://eu.idtdna.com/) accessed on 3 January 2020. A GGSGGGSG linker was inserted between the GFP and ENPL sequences. The gene encoding the GFP-ENPL fusion protein was synthetized by Bio Basic Inc., (Markham, ON, Canada) and was inserted into a pMAX plasmid (Lonza, Basel, Switzerland) for in vitro expression. The DNA sequence of the pMAXGFP-ENPL is available in Supplementary Figure S2.

### 2.10. Transfection of H9c2 and HL1 Cells with pMAXGFP-ENPL Plasmid

H9c2 and HL1 cells were cultured up to 80% confluency in antibiotic-free growth media. Before nucleofection, the cells were detached using a Trypsin EDTA solution (Merck, Darmstadt, Germany). The cells (1 × 10^7^ cells/mL) were resuspended in 14.7 µL Nucleofector Solution and 3.3 µL Supplement (SF Cell Line 4D Nucleofector^TM^ X Kit, Lonza, Basel, Switzerland) with 1 µg pMAXGFP or pMAXGFP-ENPL plasmids (in 2 µL Tris-EDTA buffer). The cells were electroporated by a 4D-Nucleofector instrument (AAF-10002X, Lonza, Basel, Switzerland), using the DS-120 program. After transfection, the cells were plated on glass coverslips (12 mm, VWR, Radnor, PA, USA) coated with 0.02% gelatin (EMD Millipore, St. Louis, MO, USA) and 5 μg/mL fibronectin (Gibco, New York, NY, USA). The efficiency of the transient transfection was examined using a Diaphot TMD Inverted Microscope (Nikon, Tokyo, Japan).

### 2.11. Detection of ENPL by Microscopy

#### 2.11.1. Membrane Staining

The HL1 cells were fixed on the third day following nucleofection. The membranes of the HL1 cells were visualized as previously described [39]. Briefly, lactadherin (Haematologic Technologies, Essex, NH, USA) was fluorescently labelled using a Cy5 Conjugation Kit (abcam, Cambridge, UK) according to the manufacturer’s instructions. Cells were fixed on the surface of chamber slides with 4% PFA in PBS for 20 min at RT. The PFA was removed with PBS containing 50 mM glycine (3 times for 5 min at RT). Fluorescent lactadherin (3 ng/μL) was applied to the fixed HL1 cells in PBS (1 h at RT). After washing with PBS (3 times for 5 min at RT), the cells with membrane-bound lactadherin were fixed again with 4% PFA in PBS and were washed with PBS containing 50 mM glycine (3 times for 5 min).

#### 2.11.2. Immunocytochemical Detection

The lactadherin-membrane-labeled cells were blocked and permeabilized with a binding buffer (BB) containing 10% FBS (Biosera, Cholet, France) and 0.5% TritonX100^®^ (Molar Chemicals, Halásztelek, Hungary) in PBS for 30 min at RT. Primary polyclonal rabbit anti-grp94 antibody was applied in 1:200 dilutions in BB for 1 h at RT (from Sigma and Merck, respectively). After washing with the BB (5 times for 5 min at RT), goat anti-rabbit-ATTO550 secondary antibodies (Sigma, Darmstadt, Germany) were used in PBS containing 1% FBS for 1 h at RT. After the washing steps (twice with PBS for 5 min, followed by twice with distilled water for 5 min), the samples were mounted with Prolong Diamond with DAPI (Invitrogen, Waltham, MA, USA) and were examined by a Leica TCS SP8 Lightning Confocal Laser Scanning microscope (Leica, Wetzlar, Germany). All antibodies are listed in Supplementary Table S3.

### 2.12. Software and Statistical Analysis

The figures were generated using GraphPad Prism 9.4.1 (GraphPad Software, Boston, MA, USA), Biorender (BioRender.com, Toronto, ON, Canada) and Microsoft Excel and PowerPoint (Microsoft, Redmond, WA, USA). For statistical analysis, the standard deviation was calculated and an unpaired Student *t*-test was applied (* *p* < 0.05).

## 3. Results

### 3.1. Characterisation of Small- and Medium-Sized Cardiomyocyte-Derived EVs under Normoxic and Hypoxic Conditions

**Figure 1:**
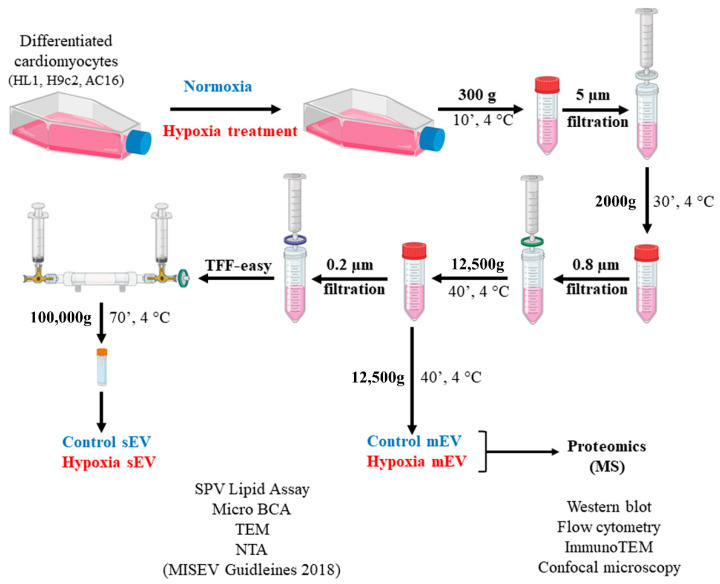
Schematic summary of the experimental approach of EV separation and detection. EVs were collected from conditioned media of control and hypoxia-treated, differentiated cardiomyocyte cells. Small (sEV)- and medium-sized EVs (mEV) were subjected to further analyses.

**Figure 2:**
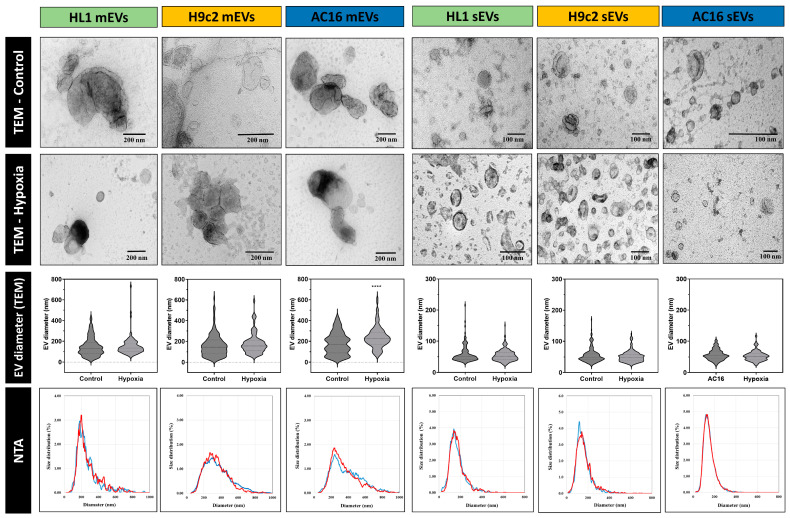
Characterization of EVs by transmission electron microscopy and nanoparticle tracking analysis. In the case of TEM analysis, a negative–positive contrasting technique with uranyl-oxalate and uranyl-acetate in methylcellulose was used. For quantitative TEM image analysis, the diameter of n = 85–150 mEVs/cell type and condition and n = 150–205 sEVs/cell type and condition were determined. **** indicates *p* < 0.0001. On the representative NTA images, blue and red curves show the size distribution of control and hypoxic-cell-derived EVs, respectively.

EVs that fell into the size ranges of small and medium EVs (sEVs and mEVs) were separated from the conditioned media of the differentiated mouse, rat and human cardiomyocyte cell lines (HL1, H9c2 and AC16, respectively). The mains steps of EV separation including the gravity-driven size filtration and differential centrifugation followed by TFF, are summarized in Figure 1. To determine the quality of the samples, the total lipid content was measured (Supplementary Figure S3). Samples that contained less than 1 µg total lipid were not included in further investigations. In the case of the AC16 and H9c2 EVs, protein to lipid (P/L) ratios were determined (Supplementary Table S4). The TEM images showed characteristic vesicular morphology and sizes. Based on the TEM images, size distributions were determined. The NTA analysis further confirmed that the separated EVs fell into the sEV and mEV size categories (Figure 2). When comparing the EVs released under normoxic and hypoxic conditions, no difference was found in the size distribution of the released EVs in most instances. The only significant difference was detected in the mEV fraction of the AC16 cells, based on the TEM analysis (Figure 2).

### 3.2. Proteomic Analysis of Cardiomycyte-Derived Extracellular Vesicle Populations

**Figure 3:**
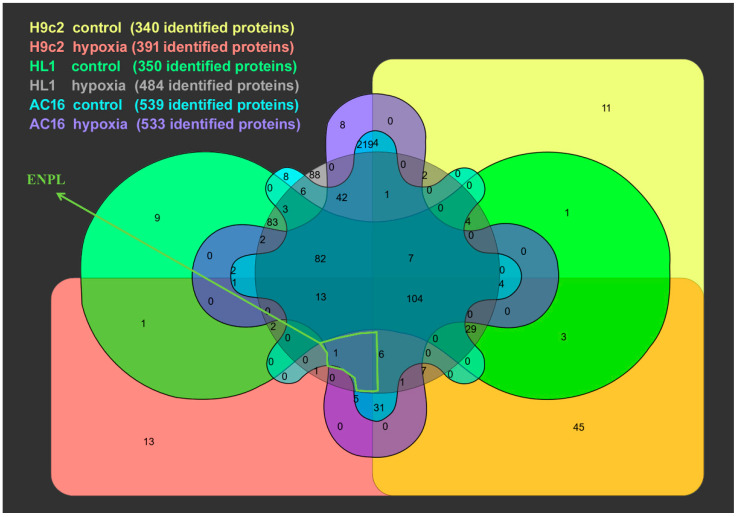
Venn diagram of proteomics. Fractions of sEV and mEVs were pooled for the analysis. The diagram indicates the number of overlapping proteins. Detailed lists of the mass spectroscopy hits can be found in the Supplementary Proteomic Data file.

**Figure 4:**
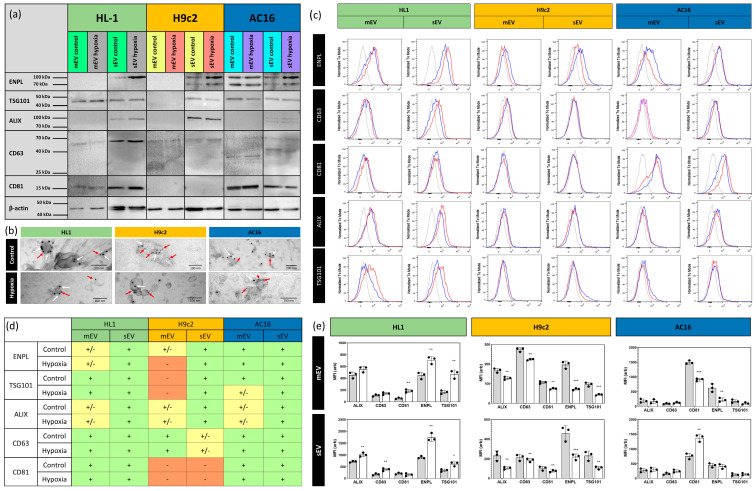
Validation of the proteomic data by different methods using purified EVs. (**a**) Western blot, (**b**) immunogold TEM and (**c**) flow cytometry. In representative flow cytometry histograms, blue curves indicate control, red curves indicate hypoxia-derived EV samples, while grey curves show antibody controls. In immunogold TEM analysis (**b**), 10 nm gold particles (white arrows) indicate ENPL, and the 5 nm gold particles (red arrows) represent CD63. Summary of qualitative analysis shows the presence (light green box) and absence (red box) of different protein markers studied by three different methods. Yellow boxes indicate markers in the case of which the presence could not be clearly confirmed (**d**). Quantification of flow cytometry is presented in panel (**e**). Results are avereges and SD of three replicates of latex bound EVs. * *p* < 0.05, ** *p* < 0.01, *** *p* < 0.001.

The separated EV fractions were subjected to proteomic analysis by MS. Lists of the protein hits in different samples and treatments can be found in the Supplementary Proteomic Data file. The number of identified proteins in the pooled mEV–sEV populations of the different cell lines under normoxic and hypoxic conditions was between 300 and 550 (Figure 3). Among the identified proteins, ENPL emerged as a protein which was present in all hypoxic cardiomyocyte-derived EV populations. Importantly, our MS analysis did not detect ENPL in normoxic HL1- and H9c2-derived EVs. ENPL was only found in normoxic AC16-derived EVs. To validate the presence of the ER resident protein ENPL in our EV preparations, qualitative immunoblotting was performed. As shown in Figure 4a, ENPL was evidently present in all hypoxic sEV preparations, while the normoxic sEVs released by the AC16, H9c2 and HL1 cell lines contained reduced, albeit detectable, amounts of ENPL. Interestingly, while both the AC16- and H9c2-cell-line-derived EVs consisted of both the 94 kDa form ENPL and the KDEL-motif truncated ENPL (approx. 75 kDa) [40], the HL1-cell EVs only contained the 94 kDa ENPL. EV markers such as TSG101, ALIX and CD63 were present in the sEV preparations, while CD81 was only present on the sEVs secreted by the AC16 and HL1 cells, and the H9c2-derived sEVs were CD81-negative. Only mEVs secreted by the AC16 cells contained ENPL. Complete Western blots are available in the Supplementary Unedited Western blots file.

Next, we tested the presence of ENPL in association with EVs using immunogold electron microscopy. As shown in Figure 4b, ENPL was detected in association with the sEVs of all three cardiomyocyte cell lines. The membranes of these sEVs were CD63-positive, excluding the ER origin of the EV membranes. Of note, the ENPL immunogold labeling was enabled by the permeabilization of the EV membranes prior to labeling. This suggests that the ENPL protein is found inside the EVs rather than on their outside surface.

Finally, we aimed at further supporting the existing data by flow cytometry. Purified sEVs and mEVs were bound onto the surface of latex beads. The histograms shown in Figure 4c correspond to that of the data obtained via immunoblotting. Flow cytometry also confirmed the presence of ENPL in association with cardiomyocyte-cell-line derived EV populations. The mean fluorescence intensities of three replicates are shown in Figure 4e. Hypoxia-dependent significant changes were found; however, there are limitations of interpreting the results of the bulk analysis of the bead-bound EVs [41].

The results of the qualitative protein analysis are summarized in Figure 4d. An EV type was determined to be positive or negative when the presence or absence of a protein was validated by all the applied qualitative methods. However, on occasion, the results were inconsistent; thus, the presence or absence could not be clearly defined.

### 3.3. Quantitative Analysis of Cardiomycyte-Derived Extracellular Vesicles

**Figure 5:**
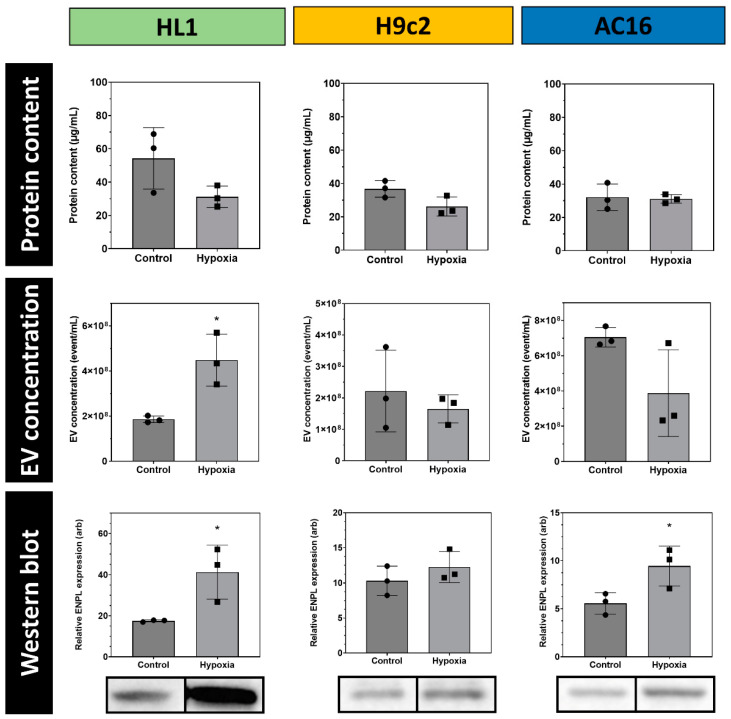
Quantitative analysis of EV release by HL1, H9c2 and AC16 cell lines. Equal amounts of cells were used during these experiments. Total protein amount was corrected with the protein content of the unconditioned media. EV concentration was determined by high-resolution flow cytometry using a shelf quenchable fluorescent dye. Quantitative Western blot data were normalized for EV concentration. Result are means and SDs of three independent biological replicates. * *p* < 0.05.

In order to eliminate the quantification uncertainties caused by EV purification, cells were grown in T25 flasks with equal seeding densities [5]. Cell- and EV-free conditioned media were used to determine the EV concentration via high-resolution flow cytometry. The total proteins were precipitated and measured by BCA. The protein content of the unconditioned medium was subtracted from the protein content. Western blotting was performed. Hypoxia did not change the protein concentration in the conditioned medium, and only the HL1-derived EV concentration changed significantly (Figure 5). Western blotting was performed in three independent biological replicates. The secreted intact (94 kDa) ENPL content was determined by densitometry and was correlated to the EV count. Under hypoxic conditions, ENPL secretion clearly and significantly increased (HL1 and AC16), and a similar trend was visible in the case of the H9c2 cells (Figure 5). Complete Western blots are available in the Supplementary Unedited Western blots file.

### 3.4. Analysis of EV Release in Transfected HL1 Cardiomyocytes

**Figure 6:**
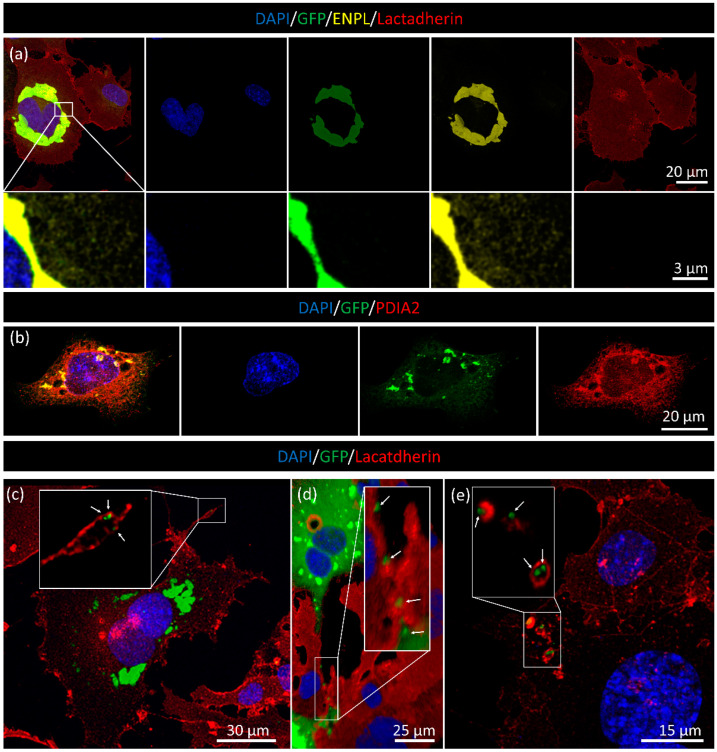
Validation of the presence of GFP-fused ENPL. (**a**) Green fluorescence (GFP), yellow (anti-ENPL with ATTO550 secondary antibody) and DAPI nuclear staining was applied. The plasma membrane was labelled by Cy5-conjugated lactadherin. The higher magnification inserts distinctly show the presence of endogenous, non-GFP fused ENPL. (**b**) The co-localization with PDIA2 ER marker protein shows the GFP signal is localized to the ER. Spotty, GFP-like accumulation is visible in the cellular extensions (**c**,**d**). White arrows point to GFP-containing EVs or EV clusters. Internalization of GFP-containing structures is visible in (**e**) and Supplementary Video S1.

Having confirmed the presence of ENPL in association with sEVs and mEVs derived from cardiomyocytes, our next step was to transfect HL1 cells with a plasmid encoding for the GFP-ENPL fusion protein. The HL1 cell line was selected because among the three cell lines we evaluated in our previous study, we found that it shows the most pronounced cardiac phenotype [24]. As shown in Figure 6a, cardiomyocytes expressed the GFP-ENPL fusion protein. Using confocal microscopy, we confirmed the GFP-ENPL expression in HL1 cells by using anti-ENPL primary and goat anti rabbit-ATTO550 secondary antibodies. The ENPL expression in the HL1 cells was visible as a brightly fluorescent perinuclear zone, which may have corresponded to the endoplasmic reticulum cisternae of the transfected cells (Figure 6a). Using a higher magnification (inserts of Figure 6a), the endogenous, GFP-free ENPL molecules were visible. Figure 6b shows co-staining with the PDIA2 ER maker. The co-localization between the green GFP and the red PDIA2 signals provides evidence for the presence of GFP-ENPL in the ER. Interestingly, some GFP-ENPL accumulation was visible in the cellular extensions (Figure 6c). These structures may have a role in the release of uptake of ENPL. In Figure 6e, plasma membrane enclosed, GFP-positive structures were identified in a non-GFP-ENPL expressing cell. We propose that a non-transfected cell is captured during the uptake of the GFP-ENPL-containing EVs by endocytosis. The details can be further followed on the Supplementary video S1 in which the 3D sections are visible with red plasma membrane staining. In Figure 6d, GFP-ENPL-containing small spherical structures (presumably corresponding to ENPL-containing EVs or EV packages) are linearly transferred between cells.

## 4. Discussion

EVs have emerged as important players in intercellular communication and in numerous homeostatic processes. Their ubiquitous nature and large heterogeneity have only been recognized in the past decades [42].

Unexpectedly, in this study, we found that an endoplasmic-reticulum-restricted chaperone protein, ENPL, is a cargo for sEVs and mEVs released by three different cardiomyocyte cell lines. We fully characterized and compared the EVs released by H9c2, AC16 and HL1 cells, the three most commonly studied cell lines in cardiovascular research.

As ER-resident proteins are often listed as contaminants of EV samples, the presence of ENPL as an EV cargo protein was unforeseen [5]. Furthermore, we addressed the question of whether ER proteins other than endoplasmin were also present in EV fractions. In the Vesiclepedia database (http://microvesicles.org/index.html accessed on 20 September 2022), searching MS-based data, we found numerous EV-associated ENPL records together with other ER resident protein entries (Supplementary Table S5). The simultaneous presence of ENPL, grp78 and calnexin in EV preparations is shown in Supplementary Figure S4. ENPL, grp78 and calnexin are often detected together in proteomics. Based on our recent findings, ENPL and the ER-resident proteins should be considered with cautiousness as non-EV contaminants of EV samples.

The MS-based proteomic data suggested that ENPL is a potential marker for the detection of cardiac injury under hypoxic conditions. The qualitative Western blotting and semiquantitative flow cytometry also showed the presence of ENPL in normoxic EV fractions. A quantitative Western blot analysis allowed for the normalization of EV quantity. Importantly, by this approach, the secretion of the intact ENPL (94 kDa) was clearly found to be increased in the EV-containing cell culture media. Of note, besides the monomeric ENPL (94 kDa), we detected other forms during Western blotting. We suggest that the low molecular weight (75 kDa) band represents the known ER-stress-induced secretory form, which lacks the KDEL motif [40], and we hypothesize that the high-molecular-weight form (approx. 140 kDa) can be a hyperglycosylated form of the molecule [43].

In confocal microscopy, we benefited from our recently developed lactadherin-based membrane-labeling approach [39] and recognized the transport of ENPL-containing particles, most likely EVs or EV clusters [11], between transfected and non-transfected HL1 cells. The suggested EV-based transport of ENPL between cardiomyocytes may drive attention to a novel aspect of the previously described cardioprotective effects of ENPL [17,19,20]. Our quantitative data refer to an increased level of ENPL in cardiomyocyte-derived conditioned medium under hypoxia. Although conclusive mechanistic evidence is still inadequate, the intracellular expression of ENPL is increased by cellular stress such as hypoxia [20]. The presence of unfolded/misfolded proteins in the ER is a proximal inducer of grp78 and ENPL [44]. Therefore, we hypothesize that some of the increasingly produced ENPL may be released by cells via EVs.

Recent studies have revealed relationships between the ER, the endoplasmic reticulum stress response and EV secretion [45]. The mechanism by which the ER-resident ENPL protein is loaded to EVs remains unknown. Recently, ER membrane contact sites with MVBs were found to be involved in RNA loading to intraluminal vesicles [46]. However, whether these ER-MVB contact sites also allow the transfer proteins, such as ENPL, to the future exosomes remains unknown.

## 5. Conclusions

Our findings presented herein shed light on the possible importance of EV-associated ER resident proteins. We provide evidence that the mEVs and sEVs of differentiated cardiomyocyte cell lines carry an ER resident protein (ENPL) as their cargo. The EV-mediated transfer of ENPL between cardiomyocytes may have a role in the maintenance of cardiac tissue homeostasis under pathological conditions.

## Data Availability

Raw data and DNA sequences are available in the Supplementary Materials.

## Supplementary Materials

The following supporting information can be downloaded at: https://www.mdpi.com/article/10.3390/membranes13040431/s1, Figure S1. Fitting of rat ENPL gene Q66HD0 and mouse P08113; Figure S2. DNA sequence of pMAXGFP-ENPL; Figure S3. Total lipid content of the sEV and mEV samples; Figure S4. ER resident protein (ENPL, grp78 and calnexin) in different EV resources based on Vesiclepedia database; Table S1. Viability% of the control and hypoxia treated cells measured by trypan blue dye exclusion test; Table S2. Viability% of cells with serum starvation compared with differentiated control cells measured by Resazurin Cell Viability Assay; Table S3. Antibodies used in our experiments; Table S4. Protein/lipid ratio of H9c2 and AC16 cardiomyocyte-derived mEV and sEV fractions; Table S5. ER resident proteins in EV, based on Vesiclepedia database; Video S1. 3D representation the endocytosis of GFP-ENPL-containing EVs; Proteomic Data File; Unedited Western blots.
