# Supplementary material for: Endoplasmin Is a Hypoxia-Inducible Endoplasmic Reticulum-Derived Cargo of Extracellular Vesicles Released by Cardiac Cell Lines
Source: Membranes (Basel). 2023 Apr 13;13(4):431. doi: 10.3390/membranes13040431 (PMC10142439; doi:10.3390/membranes13040431)

Figure S1. Fitting of rat ENPL gene Q66HD0 and mouse P08113

```
sp|Q66HD0|ENPL_RAT        MRVLWVLGLCCVLLTFGFVRADDEVDVDGTVEEDLGKSREGSRTDDEVVQREEEAIQLDG      60
sp|P08113|ENPL_MOUSE      MRVLWVLGLCCVLLTFGFVRADDEVDVDGTVEEDLGKSREGSRTDDEVVQREEEAIQLDG      60
                          ************************************************************

sp|Q66HD0|ENPL_RAT        LNASQIRELREKSEKFAFQAEVNRMMKLIINSLYKNKEIFLRELISNASDALDKIRLISL      120
sp|P08113|ENPL_MOUSE      LNASQIRELREKSEKFAFQAEVNRMMKLIINSLYKNKEIFLRELISNASDALDKIRLISL      120
                          ************************************************************

sp|Q66HD0|ENPL_RAT        TDENALAGNEELTVKIKCDREKNLLHVTDTGVGMTREELVKNLGTIAKSGTSEFLNKMTE      180
sp|P08113|ENPL_MOUSE      TDENALAGNEELTVKIKCDKEKNLLHVTDTGVGMTREELVKNLGTIAKSGTSEFLNKMTE      180
                          *******************:****************************************

sp|Q66HD0|ENPL_RAT        AQEDGQSTSELIGQFGVGFYSAFLVADKVIVTSKHNNDTQHIWESDSNEFSVIADPRGNT      240
sp|P08113|ENPL_MOUSE      AQEDGQSTSELIGQFGVGFYSAFLVADKVIVTSKHNNDTQHIWESDSNEFSVIADPRGNT      240
                          ************************************************************

sp|Q66HD0|ENPL_RAT        LGRGTTITLVLKEEASDYLELDTIKNLVRKYSQFINFPIYVWSSKTETVEEPLEEDETAQ      300
sp|P08113|ENPL_MOUSE      LGRGTTITLVLKEEASDYLELDTIKNLVRKYSQFINFPIYVWSSKTETVEEPLEEDEAAK      300
                          *********************************************************:*:

sp|Q66HD0|ENPL_RAT        EEKEEADDEAAVEEEEEEKKPKTKKVEKTVWDWELMNDIKPIWQRPSKEVEEDEYKAFYK      360
sp|P08113|ENPL_MOUSE      EEKEESDDEAAVEEEEEEKKPKTKKVEKTVWDWELMNDIKPIWQRPSKEVEEDEYKAFYK      360
                          *****:******************************************************

sp|Q66HD0|ENPL_RAT        SFSKESDDPMAYIHFTAEGEVTFKSILFVPTSAPRGLFDEYGSKKSDYIKLYVRRVFITD      420
sp|P08113|ENPL_MOUSE      SFSKESDDPMAYIHFTAEGEVTFKSILFVPTSAPRGLFDEYGSKKSDYIKLYVRRVFITD      420
                          ************************************************************

sp|Q66HD0|ENPL_RAT        DFHDMMPKYLNFVKGVVDSDDLPLNVSRETLQQHKLLKVIRKKLVRKTLDMIKKIADEKY      480
sp|P08113|ENPL_MOUSE      DFHDMMPKYLNFVKGVVDSDDLPLNVSRETLQQHKLLKVIRKKLVRKTLDMIKKIADEKY      480
                          ************************************************************

sp|Q66HD0|ENPL_RAT        NDTFWKEFGTNIKLGVIEDHSNRTRLAKLLRFQSSHHSTDITSLDQYVERMKEKQDKIYF      540
sp|P08113|ENPL_MOUSE      NDTFWKEFGTNIKLGVIEDHSNRTRLAKLLRFQSSHHSTDITSLDQYVERMKEKQDKIYF      540
                          ************************************************************

sp|Q66HD0|ENPL_RAT        MAGSSRKEAESSPFVERLLKKGYEVIYLTEPVDEYCIQALPEFDGKRFQNVAKEGVKFDE      600
sp|P08113|ENPL_MOUSE      MAGSSRKEAESSPFVERLLKKGYEVIYLTEPVDEYCIQALPEFDGKRFQNVAKEGVKFDE      600
                          ************************************************************

sp|Q66HD0|ENPL_RAT        SEKSKESREATEKEFEPLLNWMKDKALKDKIEKAVVSQRLTESPCALVASQYGWSGNMER      660
sp|P08113|ENPL_MOUSE      SEKTKESREATEKEFEPLLNWMKDKALKDKIEKAVVSQRLTESPCALVASQYGWSGNMER      660
                          ***:********************************************************

sp|Q66HD0|ENPL_RAT        IMKAQAYQTGKDISTNYYASQKKTFEINPRHPLIRDMLRRVKEDEDDKTVMDLAVVLFET      720
sp|P08113|ENPL_MOUSE      IMKAQAYQTGKDISTNYYASQKKTFEINPRHPLIRDMLRRIKEDEDDKTVMDLAVVLFET      720
                          ****************************************:*******************

sp|Q66HD0|ENPL_RAT        ATLRSGYLLPDTKAYGDRIERMLRLSLNIDPEAQVEEEPEEEPEDTTEDTTDDSEQDEEE      780
sp|P08113|ENPL_MOUSE      ATLRSGYLLPDTKAYGDRIERMLRLSLNIDPEAQVEEEPEEEPEDTSEDAEDSEQDEGEE      780
                          **********************************************:**: *..::: **

sp|Q66HD0|ENPL_RAT        TDAGAEEEEEEQETEKEPTEKDEL 804
sp|P08113|ENPL_MOUSE      MDAGTEEEEE--ETEKESTEKDEL 802
                           ***:*****  ***** ******
```

Figure S2. DNA sequence of pMAXGFP-ENPL

```
pMAXGFP-ENPL             5954 bp    DNA     circular

   1 TCAATATTGG CCATTAGCCA TATTATTCAT TGGTTATATA GCATAAATCA ATATTGGCTA
  61 TTGGCCATTG CATACGTTGT ATCTATATCA TAATATGTAC ATTTATATTG GCTCATGTCC
 121 AATATGACCG CCATGTTGGC ATTGATTATT GACTAGTTAT TAATAGTAAT CAATTACGGG
 181 GTCATTAGTT CATAGCCCAT ATATGGAGTT CCGCGTTACA TAACTTACGG TAAATGGCCC
 241 GCCTGGCTGA CCGCCCAACG ACCCCCGCCC ATTGACGTCA ATAATGACGT ATGTTCCCAT
 301 AGTAACGCCA ATAGGGACTT TCCATTGACG TCAATGGGTG GAGTATTTAC GGTAAACTGC
 361 CCACTTGGCA GTACATCAAG TGTATCATAT GCCAAGTCCG CCCCCTATTG ACGTCAATGA
 421 CGGTAAATGG CCCGCCTGGC ATTATGCCCA GTACATGACC TTACGGGACT TTCCTACTTG
 481 GCAGTACATC TACGTATTAG TCATCGCTAT TACCATGGTG ATGCGGTTTT GGCAGTACAC
 541 CAATGGGCGT GGATAGCGGT TTGACTCACG GGGATTTCCA AGTCTCCACC CCATTGACGT
 601 CAATGGGAGT TTGTTTTGGC ACCAAAATCA ACGGGACTTT CCAAAATGTC GTAATAACCC
 661 CGCCCCGTTG ACGCAAATGG GCGGTAGGCG TGTACGGTGG GAGGTCTATA TAAGCAGAGG
 721 TCGTTTAGTG AACCGTCAGA TCACTAGTAG CTTTATTGCG GTAGTTTATC ACAGTTAAAT
 781 TGCTAACGCA GTCAGTGCTC GACTGATCAC AGGTAAGTAT CAAGGTTACA AGACAGGTTT
 841 AAGGAGGCCA ATAGAAACTG GGCTTGTCGA GACAGAGAAG ATTCTTGCGT TTCTGATAGG
 901 CACCTATTGG TCTTACTGAC ATCCACTTTG CCTTTCTCTC CACAGGGGTA CCGAAGCCGC
 961 TAGCGCTACC GGTCGCCACC ATGCCCGCCA TGAAGATCGA GTGCCGCATC ACCGGCACCC
1021 TGAACGGCGT GGAGTTCGAG CTGGTGGGCG GCGGAGAGGG CACCCCCGAG CAGGGCCGCA
1081 TGACCAACAA GATGAAGAGC ACCAAAGGCG CCCTGACCTT CAGCCCCTAC CTGCTGAGCC
1141 ACGTGATGGG CTACGGCTTC TACCACTTCG GCACCTACCC CAGCGGCTAC GAGAACCCCT
1201 TCCTGCACGC CATCAACAAC GGCGGCTACA CCAACACCCG CATCGAGAAG TACGAGGACG
1261 GCGGCGTGCT GCACGTGAGC TTCAGCTACC GCTACGAGGC CGGCCGCGTG ATCGGCGACT
1321 TCAAGGTGGT GGGCACCGGC TTCCCCGAGG ACAGCGTGAT CTTCACCGAC AAGATCATCC
1381 GCAGCAACGC CACCGTGGAG CACCTGCACC CCATGGGCGA TAACGTGCTG GTGGGCAGCT
1441 TCGCCCGCAC CTTCAGCCTG CGCGACGGCG GCTACTACAG CTTCGTGGTG GACAGCCACA
1501 TGCACTTCAA GAGCGCCATC CACCCCAGCA TCCTGCAGAA CGGGGGCCCC ATGTTCGCCT
1561 TCCGCCGCGT GGAGGAGCTG CACAGCAACA CCGAGCTGGG CATCGTGGAG TACCAGCACG
1621 CCTTCAAGAC CCCCATCGCC TTCGCCGGAG GGAGTGGGGG GGGGTCCGGG CTCGAGATGC
1681 GCGTCCTGTG GGTCCTTGGA CTGTGTTGCG TGCTTCTCAC ATTCGGGTTT GTGCGAGCAG
1741 ACGACGAAGT AGATGTCGAT GGAACGGTCG AGGAAGACCT GGGTAAGTCC AGGGAGGGGT
1801 CACGGACTGA CGATGAGGTT GTGCAGCGCG AGGAAGAGGC CATACAACTC GACGGTCTGA
1861 ATGCATCCCA GATTCGGGAG CTGAGAGAGA AGTCAGAAAA GTTTGCATTT CAAGCTGAAG
1921 TTAATAGGAT GATGAAGTTG ATTATTAACT CTCTGTACAA GAATAAAGAG ATCTTTCTGC
1981 GCGAATTGAT CTCCAACGCT AGCGACGCAC TGGATAAAAT CCGGCTGATA TCACTGACCG
2041 ATGAGAATGC CTTGGCCGGT AATGAGGAAC TTACAGTAAA GATAAAGTGT GACCGAGAGA
2101 AAAATCTGTT GCACGTAACA GATACTGGTG TGGGCATGAC TAGAGAAGAA TTGGTCAAAA
2161 ACTTGGGTAC CATCGCTAAA TCTGGGACAA GCGAATTTCT TAACAAGATG ACCGAAGCAC
2221 AAGAGGACGG ACAAAGTACA AGCGAACTGA TCGGACAATT CGGGGTGGGG TTCTACAGTG
2281 CATTCCTCGT CGCAGACAAG GTGATTGTGA CGAGTAAACA CAACAACGAT ACTCAGCACA
2341 TCTGGGAATC TGACAGTAAC GAATTCAGCG TCATAGCAGA CCCCCGCGGG AATACTTTGG
2401 GGCGGGGGAC TACCATTACT CTCGTACTCA AGGAGGAAGC CTCCGATTAC CTTGAGCTTG
2461 ACACCATAAA GAATCTGGTA CGGAAGTACA GTCAATTTAT TAATTTCCCT ATCTACGTAT
2521 GGTCAAGCAA AACTGAGACG GTTGAAGAGC CTCTTGAAGA AGATGAGACA GCCCAAGAAG
2581 AAAAGGAAGA GGCAGATGAC GAGGCTGCTG TTGAGGAGGA GGAAGAGGAG AAGAAACCAA
2641 AGACCAAGAA GGTGGAAAAG ACCGTGTGGG ATTGGGAGTT GATGAATGAT ATAAAGCCCA
2701 TATGGCAGCG CCCAAGCAAA GAGGTTGAGG AGGACGAATA CAAAGCCTTT TATAAGTCAT
2761 TTAGCAAGGA GAGTGATGAC CCGATGGCTT ACATCCATTT TACTGCTGAG GGTGAGGTGA
2821 CCTTTAAAAG TATATTGTTC GTACCCACGA GTGCCCCTAG GGGACTTTTT GATGAATACG
2881 GTAGTAAGAA GTCAGACTAC ATCAAACTTT ACGTGAGACG GGTTTTTATT ACAGATGACT
2941 TCCACGATAT GATGCCTAAA TATCTGAATT TCGTGAAAGG CGTGGTCGAC TCCGATGATC
3001 TCCCCCTCAA CGTTTCTAGA GAGACACTCC AGCAGCACAA ACTTCTTAAG GTGATTCGCA
3061 AGAAGCTTGT ACGAAAGACA CTCGACATGA TTAAGAAAAT AGCAGACGAA AAATATAACG
3121 ATACTTTCTG GAAAGAGTTT GGCACAAACA TTAAGTTGGG CGTCATAGAG GACCACTCTA
3181 ACCGGACGCG GCTTGCAAAG CTCCTGCGAT TTCAGTCTTC TCACCATAGC ACCGACATAA
3241 CATCCCTTGA CCAATACGTT GAACGCATGA AAGAGAAGCA AGATAAGATT TACTTTATGG
3301 CTGGATCATC CCGGAAGGAA GCCGAGTCTT CACCCTTCGT TGAGCGACTC CTTAAGAAAG
3361 GCTATGAAGT CATCTATCTG ACAGAACCAG TGGATGAGTA CTGCATCCAG GCACTTCCGG
3421 AGTTTGACGG CAAACGCTTC CAAAATGTAG CAAAAGAAGG CGTAAAATTC GATGAGAGTG
3481 AAAAGAGTAA GGAGAGCCGA GAGGCTACCG AAAAAGAGTT TGAACCACTC TTGAACTGGA
3541 TGAAAGATAA AGCATTGAAG GACAAGATTG AAAAAGCTGT GGTAAGTCAA AGGCTGACGG
3601 AATCACCTTG TGCTCTCGTT GCATCTCAGT ACGGTTGGAG TGGAAACATG GAGAGAATTA
3661 TGAAAGCACA AGCTTATCAG ACCGGGAAGG ATATCAGTAC TAATTATTAC GCCAGCCAGA
3721 AGAAGACTTT TGAGATAAAT CCACGACATC CGCTTATTAG AGATATGCTT AGACGGGTCA
3781 AGGAGGACGA GGATGACAAG ACAGTGATGG ATCTTGCAGT GGTTCTCTTC GAAACTGCTA
```

3841 CACTCCGGTC AGGATATTTG CTGCCGGATA CGAAAGCATA TGGAGATAGG ATCGAGCGGA
3901 TGCTGCGCCT GTCACTGAAT ATTGATCCTG AGGCACAAGT TGAGGAAGAG CCCGAGGAAG
3961 AGCCGGAAGA CACGACCGAA GACACTACGG ACGACAGTGA GCAAGACGAA GAGGAGACCG
4021 ATGCCGGAGC CGAAGAAGAG GAGGAAGAAC AGGAAACCGA GAAAGAACCT ACTGAGAAAG
4081 ACGAACTTGG TGGGAGTGGG GGGGGGTCCG GGCTCGAGTG AGAGCTCGAT GAGTTTGGAC
4141 AAACCACAAC TAGAATGCAG TGAAAAAAAT GCTTTATTTG TGAAATTTGT GATGCTATTG
4201 CTTTATTTGT AACCATTATA AGCTGCAATA AACAAGTTAA CAACAACAAT TGCATTCATT
4261 TTATGTTTCA GGTTCAGGGG GAGGTGTGGG AGGTTTTTTA AAGCAAGTAA AACCTCTACA
4321 AATGTGGTAC TTAAGAGGGG GAGACCAAAG GGCGAGACGT TAAGGCCTCA CGTGACATGT
4381 GAGCAAAAGG CCAGCAAAAG GCCAGGAACC GTAAAAAGGC CGCGTTGCTG GCGTTTTTCC
4441 ATAGGCTCCG CCCCCCTGAC GAGCATCACA AAAATCGACG CTCAAGTCAG AGGTGGCGAA
4501 ACCCGACAGG ACTATAAAGA TACCAGGCGT TTCCCCCTGG AAGCTCCCTC GTGCGCTCTC
4561 CTGTTCCGAC CCTGCCGCTT ACCGGATACC TGTCCGCCTT TCTCCCTTCG GGAAGCGTGG
4621 CGCTTTCTCA TAGCTCACGC TGTAGGTATC TCAGTTCGGT GTAGGTCGTT CGCTCCAAGC
4681 TGGGCTGTGT GCACGAACCC CCCGTTCAGC CCGACCGCTG CGCCTTATCC GGTAACTATC
4741 GTCTTGAGTC CAACCCGGTA AGACACGACT TATCGCCACT GGCAGCAGCC ACTGGTAACA
4801 GGATTAGCAG AGCGAGGTAT GTAGGCGGTG CTACAGAGTT CTTGAAGTGG TGGCCTAACT
4861 ACGGCTACAC TAGAAGGACA GTATTTGGTA TCTGCGCTCT GCTGAAGCCA GTTACCTTCG
4921 GAAAAAGAGT TGGTAGCTCT TGATCCGGCA AACAAACCAC GCTGGTAGCG GTGGTTTTTT
4981 TGTTTGCAAG CAGCAGATTA CGCGCAGAAA AAAAGGATCT CAAGAAGATC CTTTGATCTT
5041 TTCTACGGGG TCTGACGCTC AGTGGAACGA AAACTCACGT TAAGGGATTT TGGTCATGCC
5101 GTCTCAGAAG AACTCGTCAA GAAGGCGATA GAAGGCGATG CGCTGCGAAT CGGGAGCGGC
5161 GATACCGTAA AGCACGAGGA AGCGGTCAGC CCATTCGCCG CCAAGCTCTT CAGCAATATC
5221 ACGGGTAGCC AACGCTATGT CCTGATAGCG GTCCGCCACA CCCAGCCGGC CACAGTCGAT
5281 GAATCCAGAA AAGCGGCCAT TTTCCACCAT GATATTCGGC AAGCAGGCAT CGCCATGGGT
5341 CACGACGAGA TCCTCGCCGT CGGGCATGCT CGCCTTGAGC CTGGCGAACA GTTCGGCTGG
5401 CGCGAGCCCC TGATGCTCTT CGTCCAGATC ATCCTGATCG ACAAGACCGG CTTCCATCCG
5461 AGTACGTGCT CGCTCGATGC GATGTTTCGC TTGGTGGTCG AATGGGCAGG TAGCCGGATC
5521 AAGCGTATGC AGCCGCCGCA TTGCATCAGC CATGATGGAT ACTTTCTCGG CAGGAGCAAG
5581 GTGAGATGAC AGGAGATCCT GCCCCGGCAC TTCGCCCAAT AGCAGCCAGT CCCTTCCCGC
5641 TTCAGTGACA ACGTCGAGCA CAGCTGCGCA AGGAACGCCC GTCGTGGCCA GCCACGATAG
5701 CCGCGCTGCC TCGTCTTGCA GTTCATTCAG GGCACCGGAC AGGTCGGTCT TGACAAAAAG
5761 AACCGGGCGC CCCTGCGCTG ACAGCCGGAA CACGGCGGCA TCAGAGCAGC CGATTGTCTG
5821 TTGTGCCCAG TCATAGCCGA ATAGCCTCTC CACCCAAGCG GCCGGAGAAC CTGCGTGCAA
5881 TCCATCTTGT TCAATCATAA TATTATTGAA GCATTTATCA GGGTTCGTCT CGTCCCGGTC
5941 TCCTCCCATG CATG

Figure S3. Total lipid content of the sEV and mEV samples were measured by sulfo-phospho vanillin (SPV) lipid assay. Red curve represents 1 μg total lipid content. Samples that contained at least 1 μg lipid were used for further investigation.

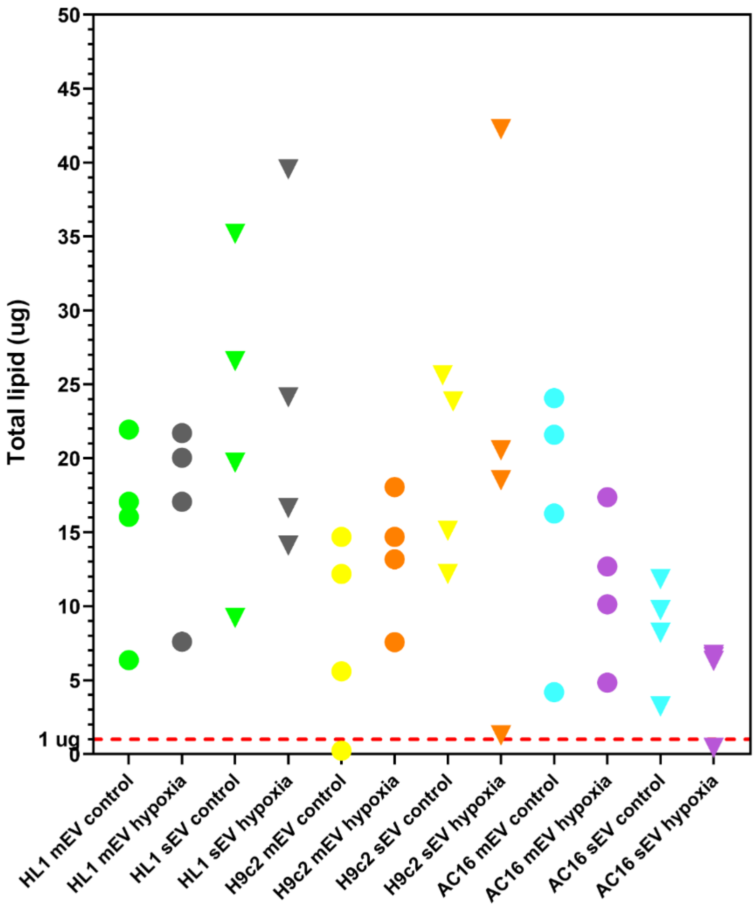

Figure S4. ER resident protein (ENPL, grp78 and calnexin) in different EV resources based on Vesiclepedia database (http://microvesicles.org/index.html). The most relevant dataset is the human (a) where at least 49 different EV type contained ER resident proteins while the mouse (b) and rat (c) database contained less available data.

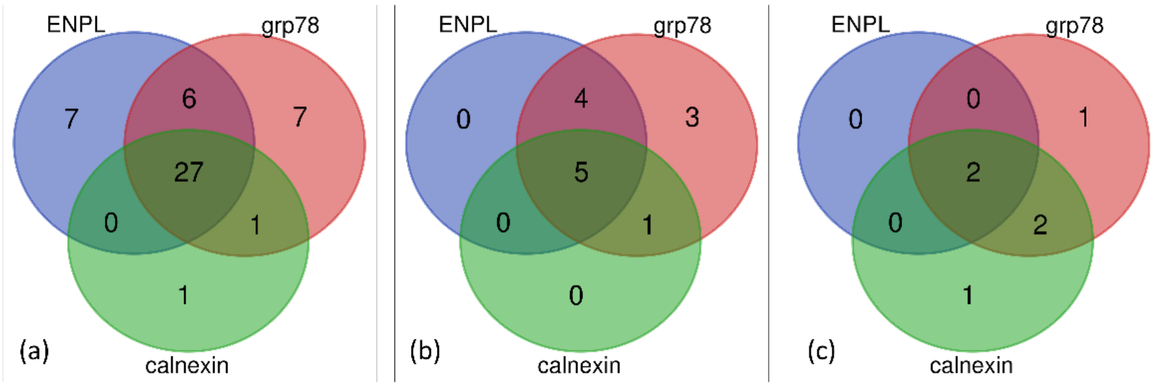

Supplement: Supplementary file 1 [file membranes-13-00431-s001.zip › Supplementary Figures.pdf]
