# Supplementary material for: Endoplasmin Is a Hypoxia-Inducible Endoplasmic Reticulum-Derived Cargo of Extracellular Vesicles Released by Cardiac Cell Lines
Source: Membranes (Basel). 2023 Apr 13;13(4):431. doi: 10.3390/membranes13040431 (PMC10142439; doi:10.3390/membranes13040431)
Supplement: Supplementary file 1 [file membranes-13-00431-s001.zip › Supplementary Tables.pdf]

**Table S1.** Viability% of the control and hypoxia treated cells measured by trypan blue dye exclusion test (Mean ± SD values are shown with n=4 biological replicates).

| **Samples** | **Viability (mean ± SD)** |
|---|---|
| HL1 control | 94.24 ± 1.91 |
| HL1 hypoxia | 94.20 ± 1.38 |
| H9c2 control | 94.56 ± 2.42 |
| H9c2 hypoxia | 93.31 ± 3.88 |
| AC16 control | 87.33 ± 6.98 |
| AC16 hypoxia | 82.92 ± 6.11 |

**Table S2.** Viability% of cells with serum starvation compared with differentiated control cells measured by Resazurin Cell Viability Assay. (Mean ± SD values are shown with n=3 biological replicates).

| **Samples** | **Viability (mean ± SD)** |
|---|---|
| HL1 | 94.65 ± 0.50 |
| H9c2 | 92.84 ± 1.99 |
| AC16 | 102.39 ± 2.17 |

**Table S3.** Antibodies used in our experiments.

| Antibody | Manufacturer | Cat.No. | Clone |
|---|---|---|---|
| rabbit polyclonal anti-CD63 (C-terminal) | Sigma/Merck | SAB2109138 | |
| mouse monoclonal anti-CD63 | SantaCruz Biotechnology | MX-49.129.5 | sc-5275 |
| rabbit polyclonal anti-CD81 | Sigma/Merck | SAB3500454 | |
| mouse monoclonal anti-CD81 | ThermoFisher | MA5-13548 | 1.3.3.22 |
| rabbit polyclonal anti-TSG101 | Sigma/Merck | HPA006161 | |
| rabbit polyclonal anti-ALIX (C-terminal) | Sigma/Merck | SAB420047 | |
| rabbit polyclonal anti-grp94 (C-terminal) | Sigma/Merck | G4420 | |
| rabbit polyclonal anti-actin N terminal | Sigma/Merck | A2103 | |
| goat-polyclonal anti-PDIA2 | Sigma/Merck | SAB2501845 | |
| goat anti-rabbit IgG-ATTO550 | Sigma/Merck | 43328 | |
| goat anti-rabbit IgG ATTO488 | Sigma/Merck | 18772 | |
| goat anti-mouse IgG CF488 | Sigma/Merck | 62197 | |
| goat polyclonal anti-rabbit IgG Fc (HRP) | abcam | ab97200 | |
| goat polyclonal anti-rabbit IgG H&L 10 nm gold pre-adsorbed | abcam | ab27234 | |
| goat polyclonal anti-mouse IgG (whole molecule) 5 nm gold pre-adsorbed | Sigma/Merck | G7527 | |
| donkey polyclonal anti-goat IgG (H+L) cross-adsorbed antibody Alexa Fluor 568 | Invitrogen | A-11057 | |

**Table S4.** Protein/lipid ratio of H9c2 and AC16 cardiomyocyte-derived mEV and sEV fractions. (Mean ± SD values are shown with n=3 biological replicates). EVs released under control and hypoxic conditions, within the same size and origin, do not differ significantly (unpaired student t-tests, p>0,05).

| **Samples** | **P/L ratio (mean ± SD)** |
|---|---|
| H9c2 mEV control | 2.55 ± 0.05 |
| H9c2 mEV hypoxia | 1.49 ± 0.94 |
| H9c2 sEV control | 0.84 ± 0.09 |
| H9c2 sEV hypoxia | 0.81 ± 0.04 |
| | |
| AC16 mEV control | 1.57 ± 0.32 |
| AC16 mEV hypoxia | 1.98 ± 0.59 |
| AC16 sEV control | 4.9 ± 1.19 |
| AC16 sEV hypoxia | 5.33 ± 0.18 |

**Table S5.** ER resident proteins in EV, based on Vesiclepedia database

| | ER resident proteins | | | | | | | | | | | |
|---|---|---|---|---|---|---|---|---|---|---|---|---|
| | ENPL (grp94/gp96/hsp90b1) | | grp78 (hspa5, BiP, MIF2) | | calnexin (CNX, IP90, P90) | | calreticulin (CRT, RO, SSA, cC1qR) | | grp170 (Hyou-1) | | Erp29 (PDI-Db, PDIA9, ErP28) | |
| | Sample source | Sample type | Sample source | Sample type | Sample source | Sample type | Sample source | Sample type | Sample source | Sample type | Sample source | Sample type |
| Homo sapiens | | | | | | | | | | | | |
| | brain cancer cells | EV (mEV+sEV) | brain cancer cells | EV (mEV+sEV) | brain cancer cells | EV (mEV+sEV) | brain cancer cells | EV (mEV+sEV) | brain cancer cells | EV (mEV+sEV) | brain cancer cells | EV (mEV+sEV) |
| | breast cancer cells | EV (mEV+sEV) | breast cancer cells | EV (mEV+sEV) | breast cancer cells | EV (mEV+sEV) | breast cancer cells | EV (mEV+sEV) | breast cancer cells | EV (mEV+sEV) | breast cancer cells | EV (mEV+sEV) |
| | bladder cancer cells | EV (mEV+sEV), EVs (sEV) | bladder cancer cells | EV (mEV+sEV), EVs (sEV) | bladder cancer cells | EV (mEV+sEV), EVs (sEV) | bladder cancer cells | EVs (sEV) | bladder cancer cells | EV (mEV+sEV), EVs (sEV) | bladder cancer cells | EV (mEV+sEV), EVs (sEV) |
| | colorectal cancer cells (CRC) | EV (mEV+sEV), mEV, exosomes (sEV) | colorectal cancer cells (CRC) | EV (mEV+sEV), mEV, exosome (sEV) | colorectal cancer cells (CRC) | EV (mEV+sEV), mEV, exosome (sEV) | colorectal cancer cells (CRC) | EV (mEV+sEV), mEV, exosomes (sEV) | colorectal cancer cells (CRC) | EV (mEV+sEV), mEV, exosomes (sEV) | colorectal cancer cells (CRC) | EV (mEV+sEV), mEV, exosomes (sEV) |
| | kidney cancer cells | EV (mEV+sEV), | kidney cancer cells | EV (mEV+sEV), | kidney cancer cells | EV (mEV+sEV), | kidney cancer cells | EV (mEV+sEV), | kidney cancer cells | EV (mEV+sEV), | kidney cancer cells | EV (mEV+sEV), |
| | lung cancer cells | EV (mEV+sEV), | lung cancer cells | EV (mEV+sEV), | lung cancer cells | EV (mEV+sEV), | lung cancer cells | EV (mEV+sEV), | lung cancer cells | EV (mEV+sEV), | lung cancer cells | EV (mEV+sEV), |
| | melanoma cells | EV (mEV+sEV), sEV | melanoma cells | EV (mEV+sEV), sEV | melanoma cells | EV (mEV+sEV), sEV | melanoma cells | EV (mEV+sEV) | melanoma cells | EV (mEV+sEV), sEV | melanoma cells | EV (mEV+sEV), sEV |
| | ovarian cancer cells | EV (mEV+sEV), exosomes (sEV) | ovarian cancer cells | exosomes (sEV) | ovarian cancer cells | EV (mEV+sEV), exosomes (sEV) | ovarian cancer cells | EV (mEV+sEV), exosomes (sEV) | ovarian cancer cells | EV (mEV+sEV), exosomes (sEV) | ovarian cancer cells | EV (mEV+sEV), exosomes (sEV) |

| | | | | | | | | | | | |
|---|---|---|---|---|---|---|---|---|---|---|---|
| prostate cancer cells | exosomes (sEV) | prostate cancer cells | exosomes (sEV) | prostate cancer cells | exosomes (sEV) | prostate cancer cells | exosomes (sEV) | prostate cancer cells | exosomes (sEV ) | prostate cancer cells | exosomes (sEV) |
| leukemia cells | EV (mEV+sEV), exosomes (sEV) , | leukemia cells | EV (mEV+sEV), exosomes (sEV) , | leukemia cells | EV (mEV+sEV), exosomes (sEV) , | leukemia cells | EV (mEV+sEV) | leukemia cells | EV (mEV+sEV), exosomes (sEV) , | leukemia cells | EV (mEV+sEV), |
| glioblastoma cells | mEV, exosomes (sEV) | glioblastoma cells | mEV, exosomes (sEV) | glioblastoma cells | mEV, exosomes (sEV) | glioblastoma cells | mEV, exosomes (sEV) | glioblastoma cells | mEV, exosomes (sEV) | glioblastoma cells | mEV, exosomes (sEV) |
| neuroblastoma cell | exosome | neuroblastoma cell | exosome | neuroblastoma cell | exosome | neuroblastoma cell | exosome | neuroblastoma cell | exosome | squamous carcinoma cells | exosomes (sEV) |
| astrocytoma cells | EV (mEV+sEV) | medulloblasto ma cells | exosome (sEV) | astrocytoma cells | EV (mEV+sEV) | astrocytoma cells | EV (mEV+sEV) | astrocytoma cells | EV (mEV+sEV) | astrocytoma cells | EV (mEV+sEV) |
| B-cells | exosomes (sEV),sEV | squamous carcinoma cells | exosomes (sEV), | B-cells | sEV | T-cells | mEV, exosomes (sEV) | B-cells | exosomes (sEV) | T-cells | exosomes (sEV) |
| T-cells | mEV, exosomes (sEV) | osteosarcoma cells | matrix vesicles (sEV) | T-cells | mEV, exosomes (sEV) | dendritic cells (THP-1 monocyte derived) | EV (sEV, mEV) | T-cells | mEV, exosomes (sEV) | dendritic cells (THP-1 monocyte derived) | EV (sEV, mEV) |
| dendritic cells (THP-1 monocyte derived) | EV (sEV, mEV) | astrocytoma cells | EV (mEV+sEV) | dendritic cells (THP-1 monocyte derived) | EV (sEV, mEV) | neutrophil granulocytes | microparticles (sEV+mEV) | dendritic cells (THP-1 monocyte derived) | EV (sEV, mEV) | neutrophil granulocytes | microparticles (sEV+mEV) |
| neutrophil granulocytes | microparticles (sEV+mEV) | B-cells | exosomes (sEV),sEV | neutrophil granulocytes | microparticles (sEV+mEV) | Monocytes (THP-1) | microparticles (mEV) | neutrophil granulocytes | microparticles (sEV+mEV) | monocytes (THP-1) | microparticles (mEV) |
| endothelial cells | microparticles (mEV+sEV), mEV | T-cells | mEV, exosomes (sEV) | endothelial cells | EV(sEV), microparticles (mEV+sEV), mEV | Macrophages (U937) | exosomes (sEV) | Monocytes (THP-1) | microparticles (mEV) | endothelial cells | microparticles (mEV+sEV), mEV |
| platelets | microparticles (mEV+sEV) | dendritic cells (THP-1 monocyte derived) | EV (sEV, mEV) | platelets | microparticles (mEV+sEV) | endothelial cells | microparticles (mEV+sEV), mEV | endothelial cells | microparticles (mEV+sEV), mEV | platelets | microparticles (mEV+sEV) |

| | | | | | | | | | | | |
|---|---|---|---|---|---|---|---|---|---|---|---|
| bronchial epithelial cells | EV (sEV) | neutrophil granulocytes | microparticles (sEV+mEV) | retinal pigment epithelial cells | exosomes (sEV) | platelets | microparticles (mEV+sEV) | platelets | microparticles (mEV+sEV) | umbilical cord mesenchymal stem cells | mEV |
| retinal pigment epithelial cells | exosomes (sEV) | Monocytes (THP-1) | microparticles (mEV) | prostate epithelial cells | exosomes (sEV) | bronchial epithelial cells | EV (sEV) | bronchial epithelial cells | EV (sEV) | neonatal myoblast cells | mEV and exosomes (sEV) |
| prostate epithelial cells | exosomes (sEV) | endothelial cells | EV(sEV), microparticles (mEV+sEV), mEV | embryonic kidney cells | exosomes (sEV) | retinal pigment epithelial cells | exosomes (sEV) | retinal pigment epithelial cells | exosomes (sEV) | thymus (human derived thymic tissue) | exosomes (sEV) |
| urothelial cells | EV (sEV) | platelets | microparticles (mEV+sEV) | umbilical cord mesenchymal stem cells | mEV | prostate epithelial cells | exosomes (sEV) | urothelial cells | EV (sEV) | malignant pleural effusion (non-small lung cancer patient derived) | exosomes microvesicles ectosomes microparticles (mEV+sEV) |
| renal proximal tubule cells | exosomes | bronchial epithelial cells | EV (sEV) | mesenchymal stem cells | mEV, exosomes (sEV) | keratinocytes | exosomes (sEV) | neonatal myoblast cells | mEV and exosomes (sEV) | carotid atherosclerotic plaque | microparticles (mEV) |
| keratinocytes | exosomes (sEV) | retinal pigment epithelial cells | exosomes (sEV) | neonatal myoblast cells | mEV and exosomes (sEV) | embryonic kidney cells | exosomes (sEV) | thymus (human derived thymic tissue) | exosomes (sEV) | plasma | microparticles (sEV+mEV) |
| embryonic kidney cells | exosomes (sEV) | prostate epithelial cells | exosomes (sEV) | thymus (human derived thymic tissue) | exosomes (sEV) | umbilical cord mesenchymal stem cells | mEV | plasma | microparticles (sEV+mEV), | urine | exosomes (sEV) |
| umbilical cord mesenchymal stem cells | mEV | urothelial cells | EV (sEV) | carotid atherosclerotic plaque | microparticles (mEV) | placental mesenchymal stem cells | exosomes (sEV) | urine | exosomes (sEV) | breast milk | exosomes (sEV) |
| placental mesenchymal stem cells | exosomes (sEV) | embryonic kidney cells | exosomes (sEV) | plasma | microparticles (sEV+mEV), | mesenchymal stem cells | mEV, exosomes (sEV) | saliva | exosomes (sEV) | | |
| mesenchymal stem cells | mEV, exosomes (sEV) | umbilical cord mesenchymal stem cells | mEV | urine | exosomes (sEV) | neonatal myoblast cells | mEV and exosomes (sEV) | breast milk | EV (sEV) | | |

| | | | | | | | |
|---|---|---|---|---|---|---|---|
| neonatal myoblast cells | mEV and exosomes (sEV) | placental mesenchymal stem cells | exosomes (sEV) | breast milk | exosomes (sEV) | thymic TEC cells) | exosomes (sEV) |
| thymic TEC cells | exosomes (sEV) | mesenchymal stem cells | mEV, exosomes (sEV) | | | thymus (human derived thymic tissue) | exosomes (sEV) |
| thymus (human derived thymic tissue) | exosomes (sEV) | neonatal myoblast cells | mEV and exosomes (sEV) | | | malignant pleural effusion (non-small lung cancer patient derived) | exosomes microvesicles ectosomes microparticles (mEV+sEV) |
| malignant pleural effusion (non-small lung cancer patient derived) | exosomes/microvesicles/ectosomes/microparticles (mEV+sEV) | thymic TEC cells) | exosomes (sEV) | | | plasma | microparticles (sEV+mEV) |
| carotid atherosclerotic plaque | microparticles (mEV) | thymus (human derived thymic tissue) | exosomes (sEV) | | | serum (TBC patient) | exosomes (sEV) |
| plasma | microparticles (sEV+mEV) | malignant pleural effusion (non-small lung cancer patient derived) | exosomes/microvesicles/ectosomes/microparticles (mEV+sEV) | | | urine | exosomes (sEV) |
| urine | exosomes (sEV) | carotid atherosclerotic plaque | microparticles (mEV) | | | saliva | exosomes (sEV) |
| saliva | exosomes (sEV) | plasma | microparticles (sEV+mEV), exosomes (sEV) | | | breast milk | exosomes (sEV) |

| | | | | | | | | | | | | |
|---|---|---|---|---|---|---|---|---|---|---|---|---|
| | seminal plasma | exosomes (sEV) | urine | exosomes (sEV) | | | | | | | | |
| | ascytes (CRC patient derived) | exosomes (sEV) | saliva | exosomes (sEV) | | | | | | | | |
| | breast milk | EV (sEV) | seminal plasma | exosomes (sEV), prostasomes (mEV+sEV) | | | | | | | | |
| | | | breast milk | EV (sEV) | | | | | | | | |
| Mus musculus | | | | | | | | | | | | |
| | breast cancer cells | exosome (sEV)s | breast cancer cells | exosome (sEV)s | breast cancer cells | exosome (sEV)s | brain cancer cells | exosomes (sEV) | breast cancer cells | exosome (sEV)s | breast cancer cells | exosome (sEV)s |
| | melanoma cells | EV (mEV + sEV) | melanoma cells | EV (mEV + sEV) | melanoma cells | EV (mEV + sEV) | breast cancer cells | exosome (sEV) | melanoma cells | EV (mEV + sEV) | melanoma cells | EV (mEV + sEV) |
| | T-cells | exosome (sEV) | T-cells | exosome (sEV) | fibroblasts | exosomes (sEV) | melanoma cells | EV (mEV + sEV) | fibroblasts | exosomes (sEV) | embryonic fibroblasts | EV (mEV+sEV) |
| | mast cells | exosome (sEV) | mast cells | exosome (sEV) | embryonic fibroblasts | exosomes (sEV), EV (mEV+sEV) | embryonic fibroblasts | (mEV+sEV) | embryonic fibroblasts | exosomes (sEV), EV (mEV+sEV) | pancreatic β-cells | exosomes (sEV) |
| | myeloid derived suppressor cells | exosome (mEV+sEV) | myeloid derived suppressor cells | exosome (mEV+sEV) | pancreatic β-cells | exosomes (sEV) | pancreatic β-cells | exosomes (sEV) | myeloid derived suppressor cells | exosome (mEV+sEV) | | |
| | osteoblasts | sEV | osteoblasts | sEV | thymus | mEV | | | | | | |
| | fibroblasts | exosomes (sEV) | fibroblasts | exosomes (sEV) | | | | | | | | |

| | | | | | | | | | | | | |
|---|---|---|---|---|---|---|---|---|---|---|---|---|
| | embryonic fibroblasts | exosomes (sEV), EV (mEV+sEV) | murine myoblasts | exosomes (sEV) | | | | | | | | |
| | thymus | mEV | murine myotubes | exosomes (sEV) | | | | | | | | |
| | | | embryonic fibroblasts | EV (mEV+sEV) | | | | | | | | |
| | | | oligodendrocytes | exosomes (sEV) | | | | | | | | |
| | | | pancreatic β-cells | exosomes (sEV) | | | | | | | | |
| | | | thymus | mEV | | | | | | | | |
| Rattus norvegicus | | | | | | | | | | | | |
| | insulinoma cell line | microparticles (mEV) | insulinoma cell line | microparticles (mEV) | insulinoma cell line | microparticles (mEV) | hepatocytes | exosomes (sEV) | NA | NA | NA | NA |
| | reticulocytes | exosomes (sEV) | hepatocytes | exosomes (sEV) | hepatocytes | exosomes (sEV) | reticulocytes | exosomes (sEV) | | | | |
| | | | retinal glial cells | exosomes (sEV) | reticulocytes | exosomes (sEV) | | | | | | |
| | | | reticulocytes | exosomes (sEV) | plasma | microparticles (sEV) | | | | | | |
| | | | plasma | microparticles (sEV) | pancreatic adenocarcinoma cells | exosomes (sEV) | | | | | | |
