# Supplementary material for: Endoplasmin Is a Hypoxia-Inducible Endoplasmic Reticulum-Derived Cargo of Extracellular Vesicles Released by Cardiac Cell Lines
Source: Membranes (Basel). 2023 Apr 13;13(4):431. doi: 10.3390/membranes13040431 (PMC10142439; doi:10.3390/membranes13040431)
Supplement: Supplementary file 1 [file membranes-13-00431-s001.zip › Unedited Western Blots.pdf]

Qualitative Western blotting with purified EV samples

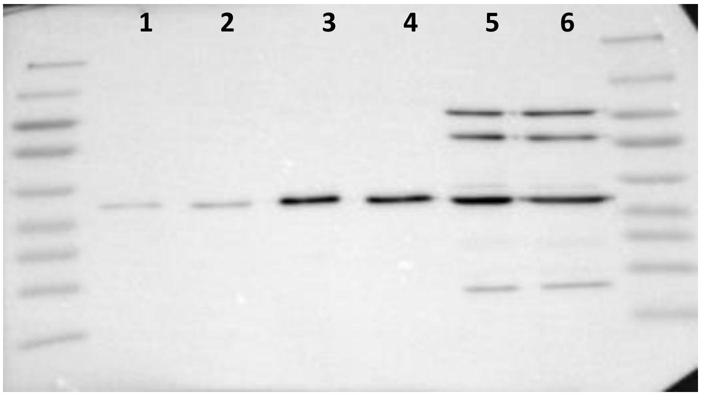

mEV

ENPL

β-actin

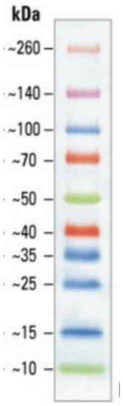

1: HL1K mEV, 2: HL1H mEV, 3: H9c2K mEV, 4: H9c2H mEV, 5:AC16K mEV, 6: AC16H mEV

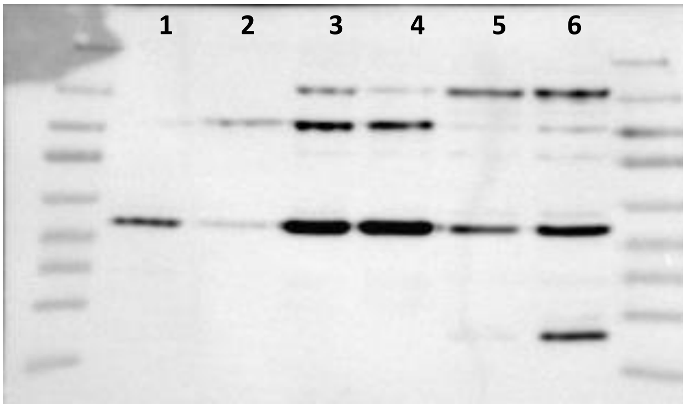

sEV

ENPL

β-actin

1: HL1K sEV, 2: HL1H sEV, 3: H9c2K sEV, 4: H9c2H sEV, 5:AC16K sEV, 6: AC16H sEV

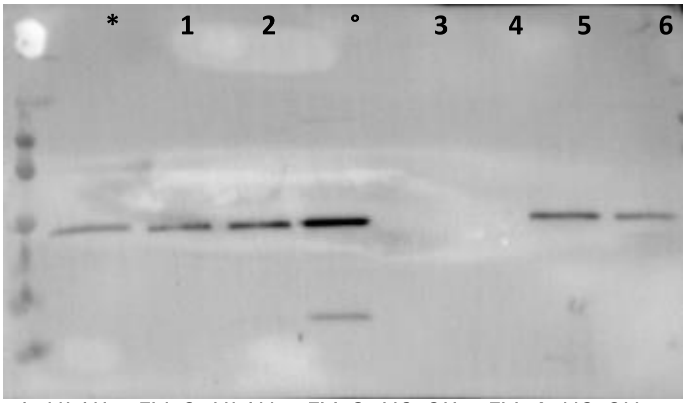

mEV

TSG101

1: HL1K mEV, 2: HL1H mEV, 3: H9c2K mEV, 4: H9c2H mEV, 5:AC16K mEV, 6: AC16H mEV, * HL1 cell lysate, ° H9c2 cell lysate

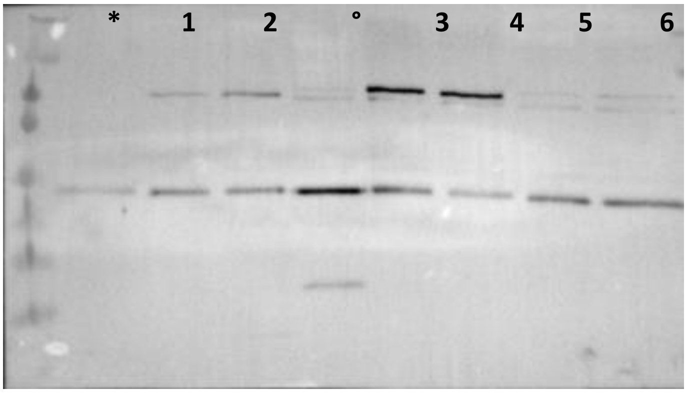

sEV

Alix

TSG101

1: HL1K mEV, 2: HL1H mEV, 3: H9c2K mEV, 4: H9c2H mEV, 5:AC16K mEV, 6: AC16H mEV, * HL1 cell lysate, ° H9c2 cell lysate

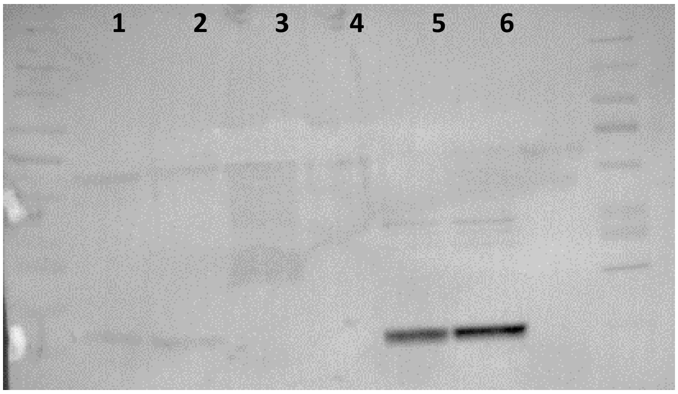

mEV

CD63

CD81

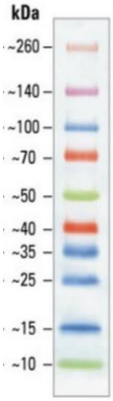

1: HL1K mEV, 2: HL1H mEV, 3: H9c2K mEV, 4: H9c2H mEV, 5:AC16K mEV, 6: AC16H mEV

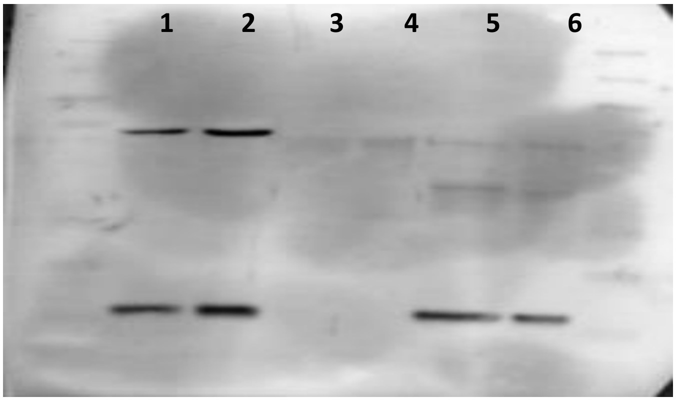

sEV

CD63

CD81

1: HL1K sEV, 2: HL1H sEV, 3: H9c2K sEV, 4: H9c2H sEV, 5:AC16K sEV, 6: AC16H sEV

mEV

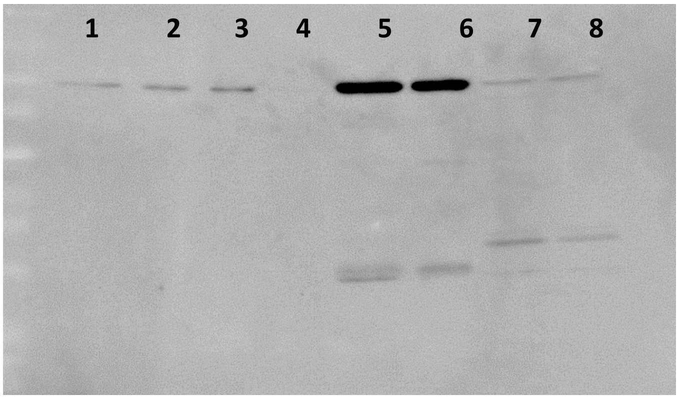

calnexin

syntenin1

1: HL1K mEV, 2: HL1H mEV, 3: H9c2K mEV, 4: H9c2H mEV, 5:AC16K mEV, 6: AC16H mEV, 7: AC16K sEV, 8: AC16H sEV

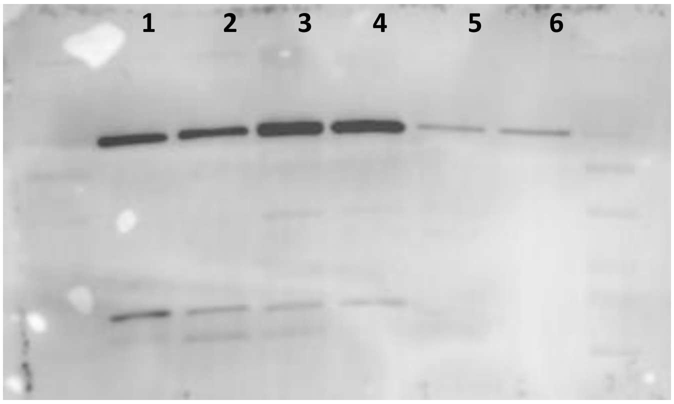

sEV

calnexin

syntenin1

1: HL1K mEV, 2: HL1H mEV, 3: H9c2K mEV, 4: H9c2H mEV, 5:AC16K mEV, 6: AC16H mEV,

Quantitative Western blotting with conditioned cell culture mediums

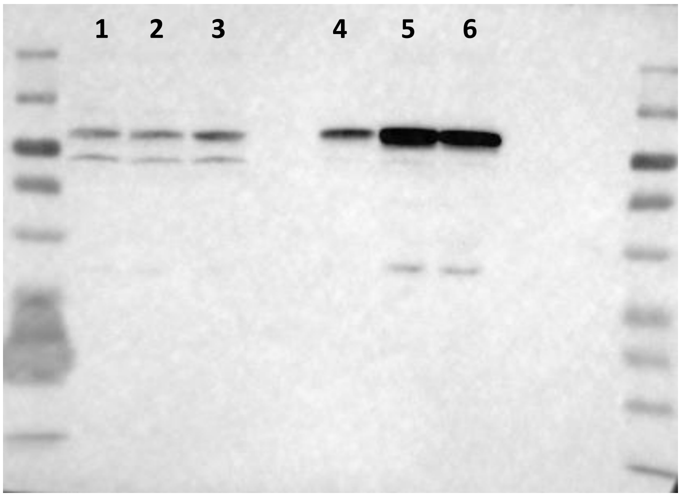

HL1 cell line

ENPL

β-actin

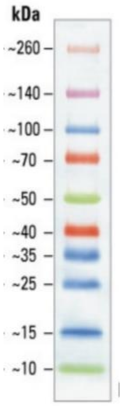

1-3: Biological replicates of control and 4-6 hypoxia treated cultures

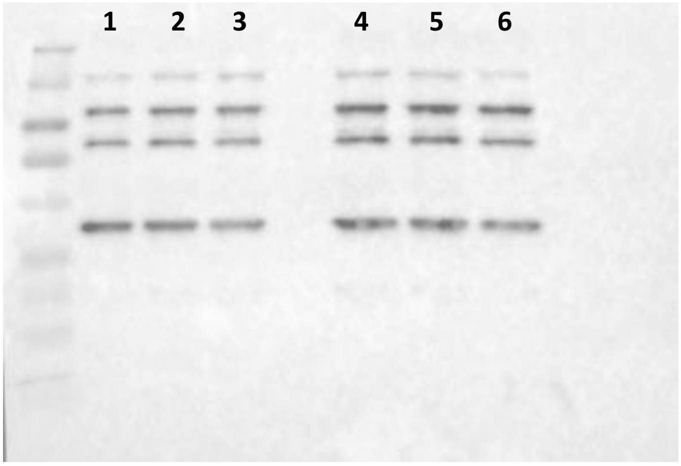

H9c2 cell line

ENPL

β-actin

1-3: Biological replicates of control and 4-6 hypoxia treated cultures

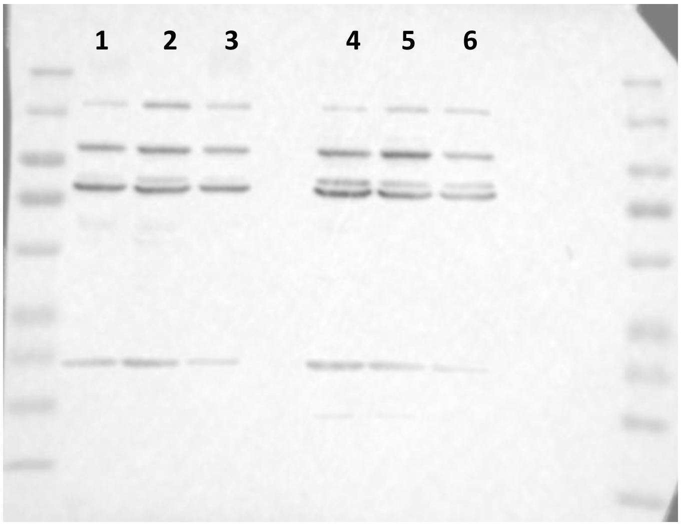

AC16 cell line

ENPL

1-3: Biological replicates of control and 4-6 hypoxia treated cultures
